# A Chronic Longitudinal Characterization of Neurobehavioral and Neuropathological Cognitive Impairment in a Mouse Model of Gulf War Agent Exposure

**DOI:** 10.3389/fnint.2015.00071

**Published:** 2016-01-12

**Authors:** Zuchra Zakirova, Gogce Crynen, Samira Hassan, Laila Abdullah, Lauren Horne, Venkatarajan Mathura, Fiona Crawford, Ghania Ait-Ghezala

**Affiliations:** ^1^The Roskamp InstituteSarasota, FL, USA; ^2^Life, Health and Chemical Sciences, The Open UniversityWalton Hall, Milton Keynes, UK; ^3^James A. Haley Veteran's HospitalTampa, FL, USA

**Keywords:** Gulf War, pyridostigmine bromide (PB), permethrin (PER), mouse model, neuropathology, neurobehavior

## Abstract

Gulf War Illness (GWI) is a chronic multisymptom illness with a central nervous system component that includes memory impairment as well as neurological and musculoskeletal deficits. Previous studies have shown that in the First Persian Gulf War conflict (1990–1991) exposure to Gulf War (GW) agents, such as pyridostigmine bromide (PB) and permethrin (PER), were key contributors to the etiology of GWI. For this study, we used our previously established mouse model of GW agent exposure (10 days PB+PER) and undertook an extensive lifelong neurobehavioral characterization of the mice from 11 days to 22.5 months post exposure in order to address the persistence and chronicity of effects suffered by the current GWI patient population, 24 years post-exposure. Mice were evaluated using a battery of neurobehavioral testing paradigms, including Open Field Test (OFT), Elevated Plus Maze (EPM), Three Chamber Testing, Radial Arm Water Maze (RAWM), and Barnes Maze (BM) Test. We also carried out neuropathological analyses at 22.5 months post exposure to GW agents after the final behavioral testing. Our results demonstrate that PB+PER exposed mice exhibit neurobehavioral deficits beginning at the 13 months post exposure time point and continuing trends through the 22.5 month post exposure time point. Furthermore, neuropathological changes, including an increase in GFAP staining in the cerebral cortices of exposed mice, were noted 22.5 months post exposure. Thus, the persistent neuroinflammation evident in our model presents a platform with which to identify novel biological pathways, correlating with emergent outcomes that may be amenable to therapeutic targeting. Furthermore, in this work we confirmed our previous findings that GW agent exposure causes neuropathological changes, and have presented novel data which demonstrate increased disinhibition, and lack of social preference in PB+PER exposed mice at 13 months after exposure. We also extended upon our previous work to cover the lifespan of the laboratory mouse using a battery of neurobehavioral techniques.

## Introduction

Studies consistently demonstrate that between 25 and 30 percent of U.S. veterans (175,000 and 210,000 of the nearly 700,000 veterans) who served in the 1990–1991 Gulf War are affected by a spectrum of multiple symptoms and continue to suffer from this persistent pattern of symptoms as a result of their wartime service (Fukuda et al., 1998; Unwin et al., 1999; Steele, 2000; N.R.C., Institute of Medicine, 2003; Abdollahi et al., 2004; Binns et al., 2008). Veterans with Gulf War Illness (GWI) exhibit persistent health issues such as fatigue, gastrointestinal problems, idiopathic pain, musculoskeletal problems, and neurological symptoms, with cognitive impairment being one of the most commonly reported symptoms (Fukuda et al., 1998; Steele, 2000; David et al., 2002; Vythilingam et al., 2005). To date, there are no effective treatments for GWI, and thus identification of biological pathways associated with long-term GWI sequelae is vital to understanding the pathogenic mechanisms of GWI and for developing novel therapies for treatment.

There are ample evidence to suggest that exposure to Gulf War (GW) agents, including pyridostigmine bromide (PB), insecticides, such as N, N-Diethyl-meta-toluamide (DEET) and pesticides, such as permethrin (PER), were key contributors to the etiology of GWI (Abdel-Rahman et al., 2004; Abou-Donia et al., 2004; Binns et al., 2008; Amourette et al., 2009; Barbier et al., 2009; Lamproglou et al., 2009; Abdullah et al., 2011, 2013). During the Persian Gulf War conflict, the carbamate compound, PB, was employed as a prophylactic agent against exposure to nerve gases such as sarin, soman, and mustard gas during time spent in foreign territory. PB reversibly inhibits the enzyme acetylcholinesterase (AChE), thus, providing enough time for clearance of the nerve gas agents that degrade the enzyme acetylcholine, before sites on acetylcholinesterase are available to bind it irreversibly. During the Persian Gulf War soldiers were supplied with PB pills in the form of a 21-tablet blister pack, with the prescribed dosage as one 30-mg tablet every 8 h (Binns et al., 2008). However, the veterans' actual exposure is unknown, since the PB pills were self-administered and there are very few examples of individual or of unit health records from the Department of Defense (Binns et al., 2008; U.S.D.o.V. Affairs, 2015). The interaction between pyrethroids such as PER and DEET has been well established in GW literature. PER is a type I synthetic pyrethroid insecticide, which exists in four different stereoisomers (Mostafalou and Abdollahi, 2013). It provides insecticidal activity for several weeks following a single application and is used to control fleas, flies, mites, and cockroaches (Chambers, 1980; Lawrence and Casida, 1983; Todd et al., 2003; Abou-Donia et al., 2004). PER functions as a neurotoxin, causing modifications of sodium channels leading to prolonged depolarization and repetitive discharges in presynaptic nerve fibers after a single stimulus (Narahashi, 1985; Bloomquist, 1996; Abou-Donia et al., 2004; Sadeghi Hashjin et al., 2010). This repetitive nerve action is associated with tremor, hyperactivity, ataxia, and convulsions. PER was utilized as a personal repellent, primarily applied on the skin and sprayed onto uniforms (Binns et al., 2008). During the GW, DEET was used as an insecticide, often in combination with other agents (i.e., PB and/or PER) by military personnel. DEET is the most common active ingredient in insect repellents and effectively “blinds” the insects by inhibiting its olfactory senses, so that the biting/feeding instinct is not triggered by the odors produced by humans or other animals (Ditzen et al., 2008). There is compelling evidence that the cause of the GWI was a complex drug interaction and that the combination of PB and PER were not the only factors (Binns et al., 2008). For instance, the combination of circulating PB has been demonstrated to further enhanced absorption of both permethrin and DEET, increasing permethrin absorption by nearly six fold (Baynes et al., 2002; Binns et al., 2008). In a different study using isolated perfused porcine skin flaps, PB administration was shown to dampen the protective inflammatory response normally stimulated by topical exposure to either permethrin or DEET, by down-regulating the production of interleukin 8 and prostaglandin E_2_ (Monteiro-Riviere et al., 2003; Binns et al., 2008). Furthermore, other chemicals such as depleted uranium, exposure to low levels of nerve gas agents including soman, sarin, and mustard gas, multiple vaccination regimes, as well as vaccinations against anthrax and botulinum, that may contribute to the etiology of GWI have also been proposed (Mahan et al., 2005; Binns et al., 2008; Shoenfeld and Agmon-Levin, 2011; Brimfield, 2012; Speed et al., 2012; Haley and Tuite, 2013; Tuite and Haley, 2013). In addition, the Gulf War scenario included low levels of sarin before and even more after the demolition of the Khamisiyah munitions depot (Haley and Kurt, 1997; Haley and Tuite, 2013). There is evidence that the severity of the GWI symptoms correlated with the times of exposure to low level of sarin recorded as alarms of detector of nerve agents (Haley and Tuite, 2013). Therefore, the mild symptoms of GWI reported here could be explained by a rather incomplete toxicological “exposure” of the mice. However, it is worth noting that the Research Advisory Committee on Gulf War Veterans' Illnesses, which extensively reviews all clinical and scientific data and literature pertinent to GWI, has suggested that PB and pesticides, such as PER, were key contributors to the etiology of GWI (Binns et al., 2008). Therefore, these agents were also chosen as they are easily amenable for experimental animal modeling in a laboratory setting, with the plan to model acute exposures to GW agents and follow the consequences of these exposures chronically.

This study aims to further develop and characterize our well-established mouse model of GW agent exposure (Abdullah et al., 2011; Zakirova et al., 2015). In particular, we wanted to further characterize neurobehavioral deficits, as these are among the main features of GWI. Thus, for this cohort of mice, we performed extensive neurobehavioral characterization from 11 days to 22.5 months post exposure to GW agents, using a battery of neurobehavioral testing paradigms. This work was done strategically in order to better study and understand disease progression, find potential biomarkers or potential therapeutic targets as well as implement treatment strategies in order to mitigate the symptoms of the disease or altogether stop the spread of the disease pathology. To the best of our knowledge, we are the first to have undertaken such an extensive chronic neurobehavioral characterization using an animal model of GW agent exposure. In addition, we have performed neuropathological studies at 22.5 months post exposure (26 months of age—close to the life-span for laboratory mice). Furthermore, as the current GWI patient population was subjected to their pathogenic exposures over two decades ago, the immediate consequences of GW agent exposure are of questionable relevance to their healthcare now. Thus, the delayed presentation evident in our model presents a platform with which to identify novel biological pathways in future studies, that may represent targets for therapeutic intervention.

## Materials and methods

### Gulf war chemical agents

PB (99.4%) was purchased from Fisher Scientific (Hanover Park, IL), and permethrin (PER) (98.3% mixture of 27.2% cis and 71.1% trans isomers) was purchased from Sigma Aldrich (St. Louis, MO). As there is no information currently available on the exact cis/trans ratio of PER that was used in the 1990–1991 Gulf War, we used this commercially available ratio since it was similar to that recommended by the World Health Organization (25% cis and 75% trans; WHO, 2009). We used 0.7 mg/kg of PB and 200 mg/kg of PER doses that have been used in previous mouse studies showing adverse behavioral or pathological outcomes (Gillette and Bloomquist, 2003; Abdullah et al., 2011; Ojo et al., 2013; Zakirova et al., 2015).

We acknowledge the limitations of this animal model of GW agent exposure, specifically, the route of administration of GW agents, PB and PER, via intraperitoneal administration. Given that, clinical literature on GWI reports that PB was taken orally by GWVs, and that PER exposure likely occurred through inhalation and/or through skin exposure. However, we would like to reiterate that this work was an extension of our previously published model. A daily consumption of 120 mg of PB for an average weight of 75 kg per individual would approximate 1.6 mg/kg. This higher dose range has been shown to inhibit AChE and activate pathways involved in long-term memory retention (Friedman et al., 1996; Vythilingam et al., 2005). However, for our current studies the dose of PB was systematically scaled down from 2 to 0.7 mg/kg in the C57BL6/J mice as higher dose(s) of PB caused insurmountable death in these mice (Dr. Ait-Ghezala, pers. comm.), this is due to the fact that the C57BL6/J mouse strain is known to exhibit cholinergic deficits (Schwab et al., 1990a,b). PER was provided to enlisted personnel as 0.5% spray, and its usage far exceeded that recommended on the PER label (Binns et al., 2008). Given the paucity of information on doses and routes (i.e., inhaled, skin absorption) of PER delivery, there is no accurate way of estimating the exact dose of PER exposure to GW veterans. Thus, we used 200 mg/kg of PER to mimic a high-level exposure that is similar to doses administered to mice in previous studies showing adverse behavioral or pathological outcomes (Pittman et al., 2003; Dodd and Klein, 2009). While we agree that investigation into the effects of different doses of GW agents in different preclinical models is critical in order to fully capture the heterogeneity of exposure and to recapitulate the clinical presentation seen in veterans with GWI, the doses for PB and PER used in this study are approximately less than one fifth and less than half of the reported LD_50_ dose for mice, respectively (Williamson et al., 1989; Chaney et al., 2002)and are therefore relevant to modeling GWI disease pathophysiology.

### Animals

All animal experiments were approved by the Roskamp Institute's Institutional Animal Care and Use Committee and conducted in accordance with the Office of Laboratory Animal Welfare and the Association for the Assessment and Accreditation of Laboratory Animal Care. Mice were purchased from Jackson Laboratories (Bar Harbor, Maine) and each mouse was individually housed in a controlled environment (regulated 14-h day/10-h night cycle) and maintained on a standard diet.

### Animal exposure

Twenty-four male C57BL6/J mice (12 weeks of age) were co-administered with either a 50 μl total volume of GW agents to a final dose of 0.7 mg/kg of PB and 200 mg/kg of PER in 100% dimethyl sulfoxide (DMSO) (exposed mice; *n* = 12), or a 50 μl volume of vehicle (100% DMSO) (control mice; *n* = 12) via intraperitoneal injection (i.p.) injection daily, for 10 days (Abdullah et al., 2011; Ojo et al., 2013). The mice were allowed to rest for an additional 10 days, and were then subjected to neurobehavioral testing. The same cohort of mice was tested repeatedly for the duration of the entire study. The Open Field Test (OFT) was conducted 11 days post exposure to GW agents. Neurobehavioral changes were previously assessed at early and late time points in other cohorts of this animal model by Abdullah et al. (2011) and Zakirova et al. (2015), in which studies cognitive impairment was observed at 5 months post exposure (see above sections). Therefore, in this chronic cohort neurobehavioral assessments were not assessed again until a much later time point. Thus, at approximately 13 months post exposure to GW agents, the mice were examined using the Elevated Plus Maze (EPM; 387 days post exposure), followed by Three-Chamber testing (395–396 days post exposure) and Radial Arm Water Maze (RAWM) testing (401–412 days post exposure). Lastly, the mice were subjected to more neurobehavioral assessments at ~22.5 months post exposure to GW agents using the Barnes Maze (BM; 674–678 days post exposure), the EPM (682 days post exposure) and the Three-Chamber Test (683–684 days post exposure). Following these evaluations the mice were euthanatized and brains submitted for neuropathological assessment.

### Behavior assessment

All behavior testing was performed during the light phase of the circadian cycle with operators blinded to the exposure assignment. All trials were recorded and analyzed with the Ethovision tracking system (Noldus, Wageningen, Netherlands). For all behavioral testing taking place between 11 days and 13 months post exposure, an *n* = 24 animals were used [controls (*n* = 12) and PB+PER mice (*n* = 12)]. For the 22.5 month time point an *n* = 18 mice were used, where controls (*n* = 6) and PB+PER mice (*n* = 12), as six controls passed away by that timepoint (from natural causes). Schematic illustrating the battery of neurobehavioral testing undertaken from 11 days to 22.5 months (684 days) post exposure to GW agents, PB+PER (Supplementary Figure 1).

#### Open field test

The OFT systematically assesses novel environment exploration, general locomotor activity, and provides an initial screen for anxiety-related behavior in rodents (Prut and Belzung, 2003; Bailey and Crawley, 2009). The OFT was conducted using a 1 m diameter arena using a single trial lasting 15 min for each mouse. The test was conducted in a brightly lit room. The OFT was conducted 11 days post exposure, at which time each animal was placed in a large open field arena for 15 min. Exploratory behavior was monitored via an overhead video camera and the video signal was analyzed using EthoVision tracking software. Key dependent measures included: cumulative distance traveled (cm) in the entire arena were recorded in order to assess locomotor activity and determine motor function, in addition, the time spent in the periphery (perimeter duration), time spent in the inner circle (s), inner circle frequency (#) defined by the number of visits into the inner circle, general mobility (s) defined by continues movement and highly mobile bouts, and general immobility (s) defined by bouts of “freezing” or not moving were investigated in order to assess anxiety-like behaviors. Dependent measures were calculated across time blocks (in 5 min increments over a 15 min period), and rates were compared across the duration of the Open Field session.

#### Elevated plus maze

The EPM is a widely used rodent behavioral test that is utilized to assess anxiety-related behavior. The EPM apparatus consists of four arms; two open, and two closed arms, the apparatus is elevated 50–70 cm from the floor. Each arm is 50 cm long and 5 cm wide, and the closed arms were shielded by 25 cm high side end walls. The four arms were linked at a central square (the junction). Briefly, similar to the study design described by Walf and Frye (2007), mice were placed at the junction of the four arms of the maze, facing an open arm, and entries/duration in each arm were recorded by both an Ethovision video-tracking system and experimenter simultaneously for 10 min. The EPM test was carried out at ~13 months (387 days) and again at ~22.5 months (682 days) post exposure to GW agents. An increase in open arm activity (duration and/or entries) reflects anti-anxiety behavior (Walf and Frye, 2007).

#### Three-Chamber Test

The Three-Chamber Test, known as the “Crawley's sociability and preference for social novelty protocol,” was used to assess sociability and social memory at ~13 months and ~22.5 months post exposure to GW agents. Testing occurred within a three-chambered box with two openings (doors) between the chambers. The two lateral compartments contain a cage comprised of a circle of vertical rods allowing sensory interaction (smell, sight, and sound) but preventing direct physical contact, preventing fighting or aggressive behaviors (Moy et al., 2004; Kaidanovich-Beilin et al., 2011). Mouse activity was recorded with the EthoVision video recording software. To begin the test, each mouse was placed in the center compartment for a 5 min habituation period, with both doors closed. To test social interaction, an unfamiliar female mouse (stranger 1) was introduced into the cage in one of the chambers, while the other compartment contained an identical empty cage. The doors were then opened from each compartment and the test mouse was left to freely explore the Three-Chamber apparatus for 10 min. For social memory testing, the test mouse was confined to the central compartment with both doors closed following the social interaction session; with the original stranger 1 mouse still in place, a second novel unfamiliar female mouse (stranger 2) was introduced in the previously empty cage after which the doors were again opened. The test mouse was then left to freely explore the Three-Chamber apparatus for 10 min. The apparatus was cleaned with 70% ethanol between each test mouse. Measures included: total distance moved, time spent in the compartment containing stranger 1 and the empty cage for the social interaction phase, and time spent in the chamber containing stranger 2 and stranger 1 for the social memory phase of the test. We acknowledge the limitations of this experimental design in regards to the Three Chamber Test: due to the aggressive nature of the male C57BL6/J mice, all mice were signally housed and therefore, social deprivation was an inherent feature of this experiment.

#### Radial arm water maze

The RAWM was used to test spatial memory and learning, and the testing was conducted ~13 months post exposure to GW agents. The RAWM is a circular pool that contains six swim arms extending into an open central area, with a hidden escape platform submerged at the end of one arm (fixed between trials) known as the goal arm. Each mouse was trained for 5 days, which included nine trials per day, with each trial lasting for 60 s. Throughout the RAWM training days, reference, and working memory errors were assessed. Reference memory reflects learning that is based on trial-independent procedural aspects of the task, where the mouse navigates using spatial cue locations, while working memory is based on trial-dependent and describes the ability of the mouse to retain this trial-dependent information, for instance the arms previously visited, in his memory (Frick et al., 1995; Shukitt-Hale et al., 2004) (Luine et al., 1998). In this experiment, reference memory errors were assessed by measuring the number of incorrect arm entries before reaching the goal arm where the escape (hidden) platform was located. While, working memory errors were assessed by measuring the number of incorrect arm entries such as entering the same arm twice or visiting an arm out of sequence. Each wrong arm entry was scored as “error.” After the completion of the five trainings, mice that have correctly learned the location of the escape platform in RAWM will exhibit a performance error of 1 or less. The mice that have failed to learn well will exhibit 3–5 errors throughout the training sessions without apparent improvement over trials. Latency to reach the goal arm was also recorded during the trials and was analyzed per block (Diamond et al., 1999). The RAWM has been shown to be a sensitive measure for detecting memory deficits and to discriminate between mice that learn well from those that learn poorly (Diamond et al., 1999; Alamed et al., 2006).

#### Barnes maze

The BM protocol was used to assess spatial memory and learning at ~22.5 months (674–678 days) post exposure to GW agents (Barnes, 1979; Sunyer et al., 2007). The BM acquisition trials were conducted 674–678 days post-exposure, during which time four trials were conducted per mouse per day for 4 days. In order to assess short-term spatial working memory, a single probe trial was carried out 679 days post-exposure. Briefly, during the 4 days of acquisition trials (four trials/mouse/day), each mouse was placed in the middle of the maze and allowed to explore the maze for a fixed interval of 180 s. The escape box was placed underneath the Target Hole (TH), and the mouse was allowed to escape and rest in the chamber for the duration of the acquisition trial. If the mouse did not find the TH, then the mouse was guided to the TH, allowed to enter the escape box and remained in the box for 60 s. On the probe trial day (5th day), the escape box was removed, and each mouse was then placed in the middle of the maze and allowed to explore the maze for a fixed interval of 90 s. The number of nose pokes in the TH (frequency), the number of nose pokes into the holes other than the TH (primary error rate) and the TH duration [the time(s) the mouse spent at the TH], as well as the distance to TH (cm) (distance traveled to reach the virtual TH), were among the dependent variables that were measured during the experiment as outcome factors.

### Statistical analyses for behavioral testing

The following behavioral analyses for the Chronic Cohort were carried out using JMP 11.0 software (SAS, Cary, NC). The OFT was carried out 11 days post exposure to GW agents. The Shapiro-Wilk test was used to assess for normality (normally distributed data). All dependent variables were normally distributed, thus, a linear regression model was employed to examine the following dependent variables: Cumulative Distance Moved (cm), Perimeter Duration (s), Mobile Duration (s), and Immobile Duration (s) for the OFT. EPM data were normally distributed when the mice were examined at 13.5 months and 22.5 month time points post exposure, thus, One-way analysis of variance (ANOVA) was used for the following dependent variables: time spent in the closed arms (s), time spent in the open arms (s), time spent in the center (s) (junction between the closed and the open arms), number of visits to the closed arms (#), number of visits to the open arms (#), number of visits to the center (#), cumulative distance traveled (cm), and velocity (cm/s). Data from the Three Chamber Test were normally distributed, therefore, matched pairs analysis which compares the means between two or more correlated variables and assesses the differences, was used for the following dependent variables: Social Interaction, the proximity to either the stranger mouse (stranger 1) or the empty cage (s); Social Memory, the proximity to either the familiar mouse (stranger 1) or the novel mouse (stranger 2); Sociability, the number of visits to either stranger 1 or the empty cage (#); Social Novelty (#), the number of visits to either familiar mouse or the novel mouse. Proximity to either the stranger mouse (stranger 1) or the empty cage (social interaction) data was transformed using square root to achieve normal distribution for both 13.5 and 22.5 month time points. BM testing was conducted ~22 months post exposure to GW agents. For the BM acquisition trials conducted at 674–678 days post exposure to GW agents, the following dependent variables: Average Distance to TH (cm), Cumulative Distance to TH (cm), Velocity (cm/s), Frequency (#), and Duration at TH (s) were normally distributed, thus, a Multivariate Model with Repeated Measures was used. However, when dependent variables, such as Latency (s), were not normally distributed, matched pairs with Wilcoxon sum rank testing was used to assess differences between control and exposed mice during BM acquisition trials. When examining the probe trial data, the following dependent variables: Average Distance to TH (cm), Cumulative Distance to TH (cm), Velocity (cm/s), Primary error rate (#)- were normally distributed but had unequal variances, thus, Welch's *t*-test was used. However, when dependent variables, such as Latency (s), Duration at TH (s), and Frequency (#), were not normally distributed, Wilcoxon sum rank testing was used to assess differences between control and exposed mice during the BM probe trial.

The RAWM was conducted at ~13 months post exposure to GW agents. RAWM data were analyzed using SPSS 21.0 (IBM corp., Armonk, NY). A mixed linear model (MLM) regression was employed to examine the independent effects of exposure and time, and any potential interactions between them, followed by *post-hoc* comparison of estimated marginal means using least significant difference (LSD) for control vs. PB+PER exposure for each day separately, for the following dependent variables: cumulative distance traveled (cm), platform latency (s), platform duration (s), and number of errors (#), as well as goal arm latency (s), goal arm frequency (number of visits to the goal arms) (#), and velocity (cm/s). The MLM-based regression analysis approach is generally considered advantageous over other ANOVA due to its flexibility to accommodate fixed and random effects of the independent variables as well as its ability to incorporate dichotomous, continuous, and categorical variables (Katz, 2011). However, if the data were not normally distributed, a generalized linear model (GLM) was used to perform the analyses and evaluated non-parametric dependent variables using the Wald test. When examining the probe trial data, the dependent variables: duration (time spent) at the goal arms (s), number of reference memory errors (#), and the number of working memory errors (#), were not normally distributed, therefore GLM was used to perform was used to perform the analyses and evaluated non-parametric dependent variables using Tweedie with log link the analyses. Statistical significance was set at the alpha 0.05 level for all statistical analysis.

GraphPad Prism 6 software (La Jolla, CA) was used to generate the graphs/diagrams of behavioral data.

### Immunohistopathological procedures

The left hemisphere from all animals was immersed in 4% Paraformaldehyde (PFA) for 24 h and paraffin embedded. Six μm thick sagittal sections were deparaffinized and rehydrated in an ethanol gradient before the start of each procedure. Three sets of 1 in 10 serial sagittal brain sections per animal, and a minimum of four animals per group from the Chronic Cohort, were used for each study.

#### Histological staining

Nissl staining was performed in order to examine the morphology and pathology of neuronal tissue. The *in situ* cell death detection kit (Roche Diagnostics, Indianapolis, IN) was used for terminal deoxynucleotidyl transferase dUTP nick end labeling (TUNEL) staining, following manufacturer's instructions. Labeling was performed with 3,3′ Diaminobenzidine (DAB) as the chromogen. Bielschowsky's silver staining was also used to check for the presence of degenerating neurons and damaged axons, as per manufacturer's instructions (HitoBiotec Inc., Wilmington, DE).

#### Immunohistochemical staining

Glial fibrillary acidic protein antibody (GFAP, 1:10000, Dako, Carpinteria, CA) and ionized calcium binding adaptor molecule 1 antibody (IBA-1, 1:1000, Abcam, Cambridge, MA) were used to stain for astrocytes and for both activated and resting microglia/macrophages, respectively. Doublecortin (DCX, 1:1,000, Santa Cruz Biotechnolgy, TX) was used to stain for newly born neurons. Pre-synaptic vesicles were detected using anti-synaptophysin antibody (SYP, 1:400, Abcam, Cambridge, MA). Tissue sections were subjected to heat-induced antigen retrieval using target retrieval solution, citrate buffer pH 6 (Dako, Carpinteria, CA) for IBA-1 and SYP IHC procedures. Endogenous peroxidase activity was quenched with H_2_O_2_treatment (0.3% in water). Each section was rinsed and incubated with the appropriate blocking buffer (DAKO Serum Free Protein Block), before the appropriate primary antibody was applied and the sections left to incubate overnight at 4°C. The diluted biotinylated secondary antibody from the ABC Elite Kit (VECTASTAIN Elite ABC Kit, Vector Laboratories, Burlingame, CA) was then applied. The stain was developed using DAB peroxidase substrate solution and counterstained with hematoxylin. All IHC slides were dehydrated through the ethanol gradient, treated with xylene, and subsequently coverslipped with permanent mounting medium. Both microscopy and quantification were performed with the operator blinded to the exposure assignment.

#### Immunohistochemical image analysis

Briefly, non-overlapping RGB (red, green, blue) images were digitally captured within the defined areas of interest (hippocampi, including the dentate gyri, and the CA3 regions, and the cerebral cortices from exposed and control mice, respectively) and were then optically segmented and analyzed as previously described by Schindelin et al. (2012) using the FIJI open-source platform for biological image analysis (http://fiji.sc/Fiji).

### Statistical analysis for immunoreactivity of stained tissues

The immunoreactivity (percent area) of tissue stained with DAB was calculated by dividing the obtained mean RGB value (of segmented profiles) by the total RGB value per defined field, multiplied by 100. Data were separately plotted as the mean percentage area of immunoreactivity per field (denoted “% Area”) ± SEM for each region and grouping. We manually counted the number of DCX+ cells in the dentate gyri of exposed and control animals, as previously described by Ojo et al. (2013). One-way ANOVA was used if the outcome variable was normally distributed, otherwise non-parametric testing such as Wilcoxon sum rank testing was utilized. Statistical analysis was performed using JMP 11.0 (SAS, Cary, NC).

GraphPad Prism 6 software (La Jolla, CA) was used to generate the graphs/diagrams of histological and immunohistochemical data.

### Quantification of ACh levels from brain homogenates

Determination of acetylcholine (ACh) levels was performed as previously described by Ojo et al. (2013) and Zakirova et al. (2015) with slight modifications. Briefly, 10 μl of brain homogenate (homogenized in 4X v/w in MS grade water with no protease/phosphatase inhibitor cocktail) from the right hemisphere was combined with 10 μl of 1.5 μg/mL ACh-D4 (CDN Isotopes, Quebec, Canada) internal standard in 100% Mass Spectrometry (MS) grade acetonitrile (ACN). This was followed by an addition of 80 μl of ice-cold MS grade ACN to each sample, which was then centrifuged for 10 min at 15,800 × g. The supernatant for each sample was subsequently transferred to individual glass vials (Restek, Pennsylvania, US) and used for MS analysis.

ACh levels were analyzed by direct infusion MS. Chip-based nanospray was used to introduce each sample into an LTQ-Orbitrap mass spectrometer (Thermo, Waltham, MA, USA) via a Nanomate Triversa (Ithaca, NY, Advion). Acetylcholine and ACh-D4 precursor molecular ions, 146.12 and 150.14 m/z, respectively, were isolated simultaneously (isolation width = 10) in the ion trap and fragmented using higher energy collisional dissociation (HCD) in the C-trap (relative collision energy = 77). Fourier transform mode (FTMS) at 100,000 resolution (m/z = 400) was used to acquire MS/MS spectra. Injections lasted for 30 s, and five technical replicates were performed for each sample. Acetylcholine levels were calculated from the peak height ratios of D_0_ and D_4_ fragment ions (87.04 m/z for ACh-D_0_ and 91.07 m/z for ACh-D_4_) for each of the five replicates. Concentrations of ACh levels in each sample were calculated in reference to the concentration of the ACh-D_4_ internal standard, which were then normalized to the total protein concentration of each respective sample. Statistical analyses of ACh data were performed with MLM regression analyses using SPSS 21.0 (IBM corp. Armonk, NY).

## Results

### Neurobehavioral examination

**Figure 1:**
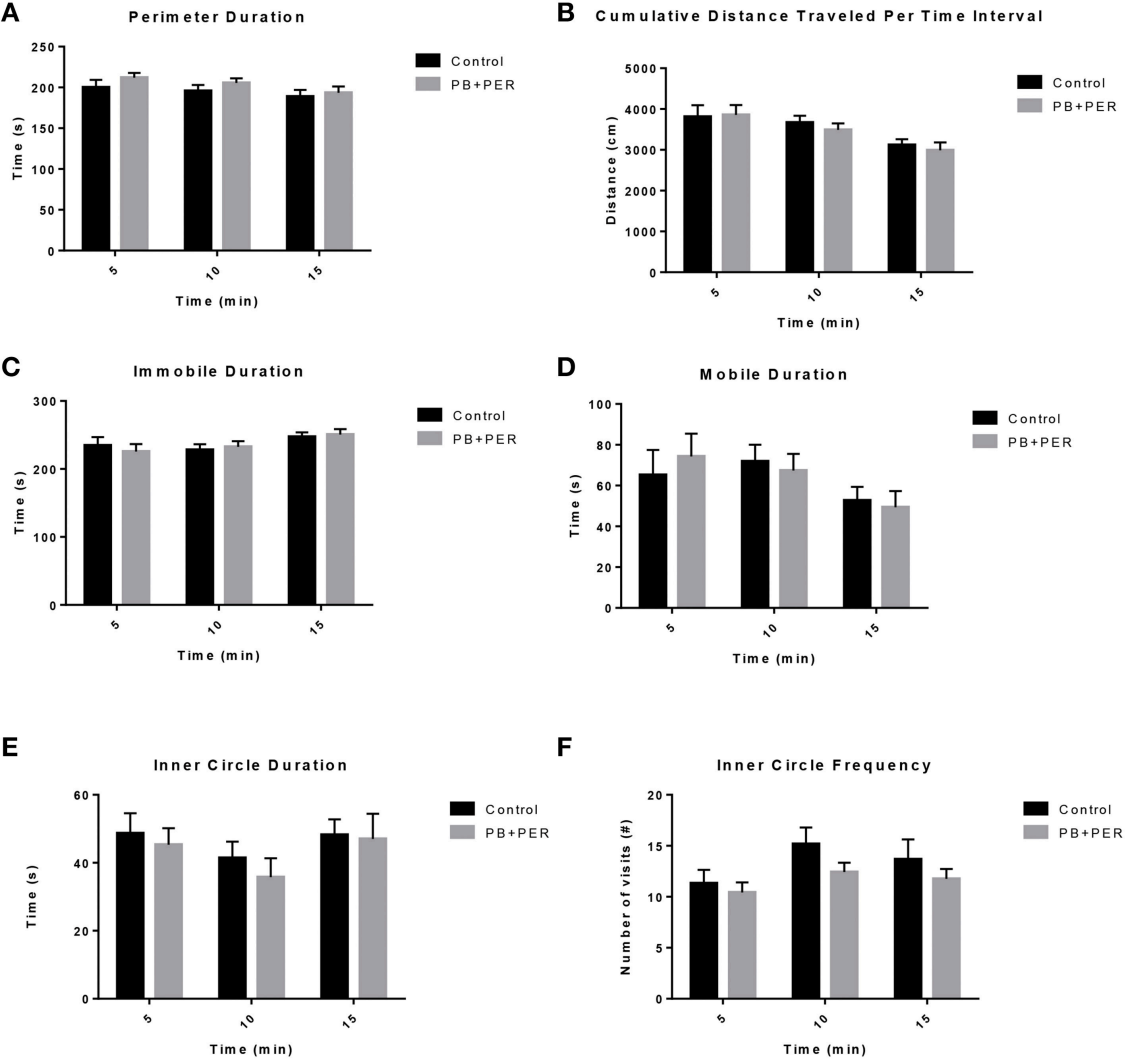
**No locomotor impairment and anxiety-like behaviors were observed at 11 days-post acute exposure to PB+PER. (A)** Perimeter Duration (s), **(B)** Cumulative Distance Traveled Per Time Interval (cm), **(C)** Immobile Duration (s), **(D)** Mobile Duration (s), **(E)** Inner Circle Duration (s), and **(F)** Inner Circle Frequency (#) were examined for exposed and control mice during the Open Field Test.

The OFT revealed that there was no locomotor impairment, nor any anxiety related behaviors (Figure 1; Supplementary Table 1) observed in the PB+PER mice when compared to control mice, 11 days post exposure to GW agents.

**Figure 2:**
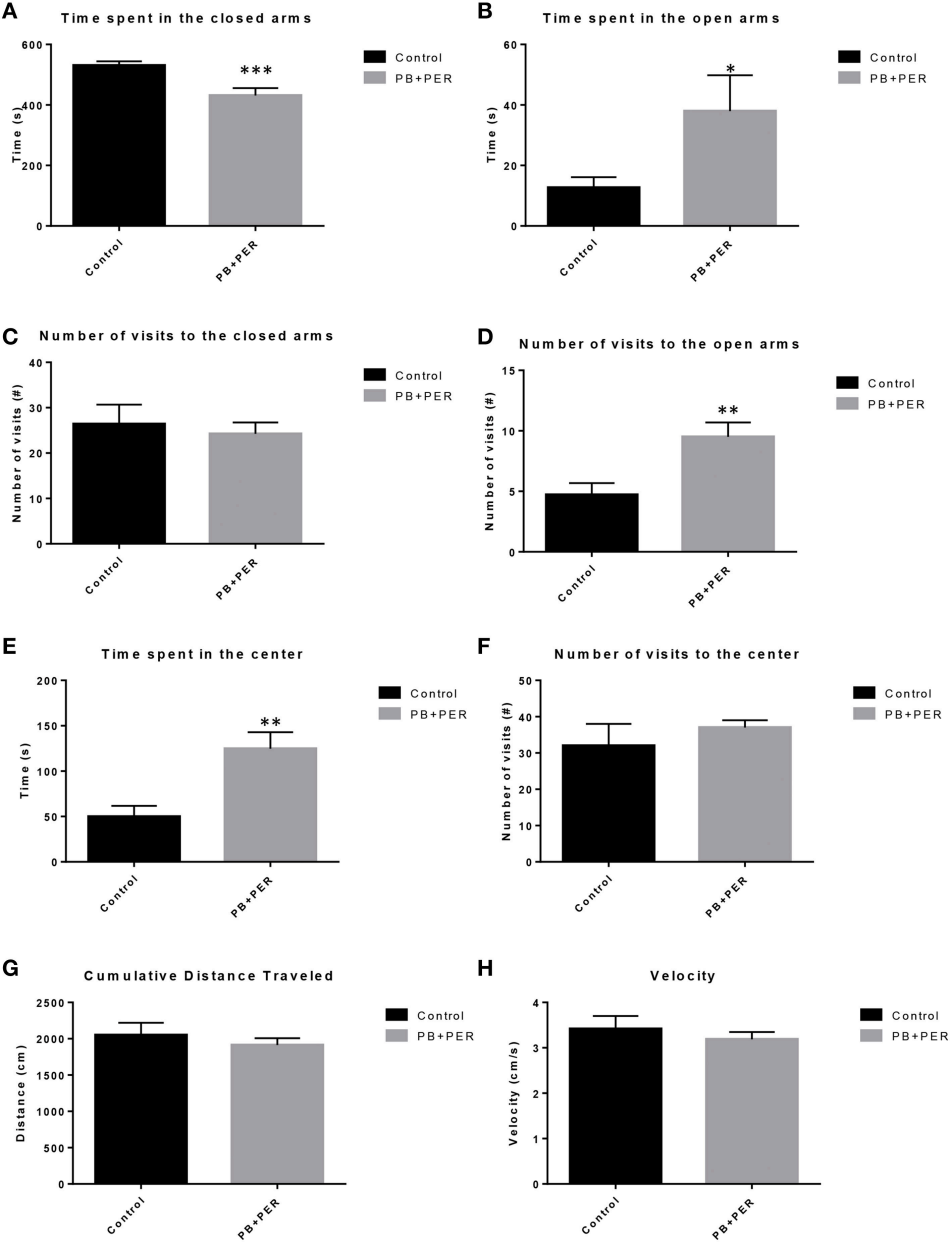
**PB+PER exposed mice demonstrated disinhibition, 13 months post exposure, as revealed by Elevated Plus Maze testing**. EPM testing revealed that PB+PER mice spent significantly less time in the closed arms **(A)**, and a significantly larger proportion of time in the open arms **(B)** as compared to their controls. No differences were noted between the two groups when examining **(C)** number of visits to the Closed Arms (#). PB+PER mice visited the open arms (#) more times as compared to the control mice **(D)**. In addition, exposed mice spent more time in the center as compared to the control mice **(E)**. Therefore, the exposed mice exhibited reduced anxiety-like behaviors. No differences were noted between the two groups when examining the number of visits to the center area (the junction between the closed and the open arms) **(F)**. Furthermore, EPM testing revealed that there were no differences between exposed and control mice when examining **(G)** Cumulative distance moved (cm) and **(H)** Velocity (cm/s). ^*^p < 0.05; ^**^p < 0.01; ^***^p < 0.005.

**Figure 3:**
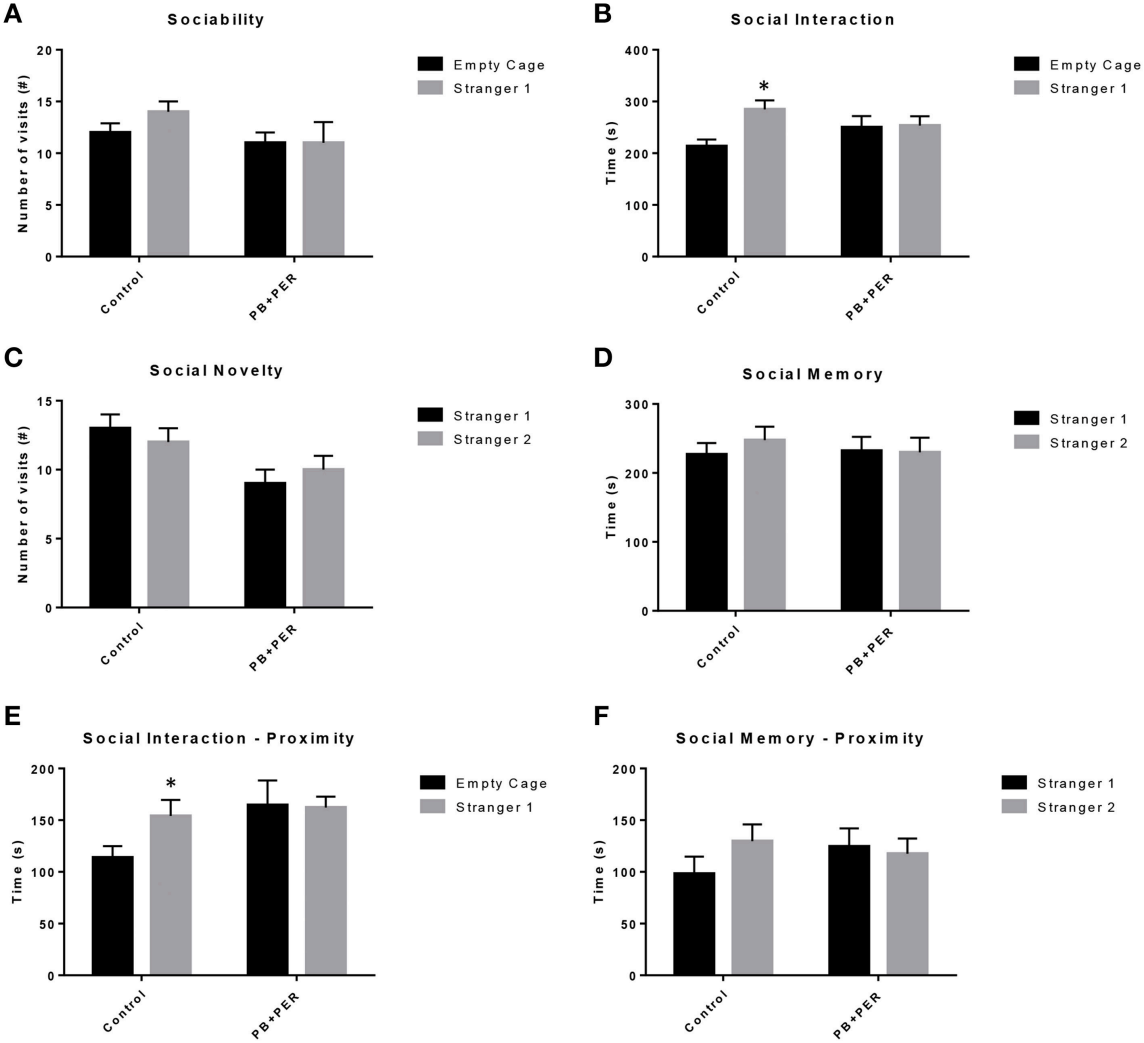
**PB+PER exposed mice showed a lack of social preference at 13 months post exposure to GW agents**. When examining **(A)** Sociability, the number of visits to either the empty cage or stranger 1, the control mice exhibited a slight trend toward an increased number of visits to the stranger 1 mouse vs. the empty cage, although it did not reach statistical significance, it did exemplify normal behaviors, demonstrating preference for the novel mouse (stranger 1). However, the PB+PER exposed mice showed a lack of social preference, by visiting the empty cage and the stranger 1 an equal number of times. When examining **(B)** Social Interaction, the time (s) the animals spent with either the empty cage or stranger 1, the control mice spent significantly more time with stranger 1 as compared to the empty cage. However, the PB+PER exposed mice showed a lack of social preference spending an equal amount of time with either the empty cage or the stranger 1. When examining **(C)** Social Novelty, the number of visits to either stranger 1 (familiar mouse) or stranger 2 (novel mouse), the control mice did not indicate a strong preference for one or the other, likewise, no differences in social novelty were evident when examining PB+PER exposed. In addition, when examining **(D)** Social Memory, as indicated by the time spent with either stranger 1 or stranger 2, the control mice appeared to spent slightly more time with the stranger 2, however, those differences did not reach statistical significance, the PB+PER exposed mice did not show a strong preference for the novel mouse (stranger 2) over the familiar mouse (stranger 1). When examining **(E)** Social Interaction—Proximity (s), the control mice showed a statistically significant preference to spent more time with stranger 1 as compared to the empty cage, demonstrating normal healthy social behaviors. However, the PB+PER mice a lack of social preference, demonstrated by a similar amount of time spent between stranger 1 and the empty cage. When examining **(F)** Social Memory—Proximity (s), the control mice showed a trend to spend more time with stranger 2 as compared to stranger 1, indicative of normal healthy social behaviors, although those differences did not reach statistical significance. However, the PB+PER mice once again showed a lack of social preference, demonstrated by an similar amount of time spent between stranger 1 and stranger 2. ^*^p < 0.05.

EPM conducted at 13 months post exposure revealed that exposed mice spent significantly less time in the closed arms (*F* = 5.10, *DF* = 1, *p* = 0.004; Figure 2A) and a significantly larger proportion of time in the open arms (*F* = 6.67, *DF* = 1, *p* = 0.03; Figure 2B) as compared to their controls. Furthermore, exposed mice visited the open arms more times as compared to the control mice (*F* = 9.30, *DF* = 1, *p* = 0.01; Figure 2D), denoting a reduction of thigmotaxis, which involves avoidance of open areas. In addition, exposed mice spent more time in the center as compared to the control mice (*F* = 13.02, *DF* = 1, *p* = 0.006; Figure 2E). Therefore, the exposed mice exhibited reduced anxiety-like behaviors. No differences were noted between the two groups when examining number of visits to the closed arms (#) (Figure 2C; *F* = 0.13, *DF* = 1, *p* = 0.73) and the number of visits to the center area (the junction between the closed and the open arms; *F* = 0.28, *DF* = 1, *p* = 0.61; Figure 2F). Indicating that once the PB+PER mice arrive to center they remain there for a larger duration, even though the number of visits to the center area is not different as compared to controls. EPM testing revealed that there were no differences between PB+PER exposed and control mice when examining cumulative distance traveled (*F* = 0.33, *DF* = 1, *p* = 0.57) and velocity (cm/s) (*F* = 0.34, *DF* = 1, *p* = 0.57; Figures 2G,H, respectively), indicating that these parameters were not influencing entry behavior. The EPM test was followed by the Three Chamber Test to assess social interaction behavior (general sociability) and interest in social novelty in exposed mice and their controls at the 13 month post exposure time point. When examining Sociability, the number of visits to either the empty cage or stranger 1, the control mice exhibited a slight trend toward an increased number of visits to the stranger 1 mouse vs. the empty cage, although it did not reach statistical significance (*t*-ratio = −1.64, *DF* = 9, *p* = 0.14; Figure 3A), it did exemplify normal behaviors, demonstrating preference for the novel mouse (stranger 1). However, the PB+PER exposed mice showed a lack of social preference, by visiting the empty cage and the stranger 1 an equal number of times (*t*-ratio = 0, *DF* = 11, *p* = 1.0; Figure 3A). When examining Social Interaction, the time (s) the animals spent with either the empty cage or stranger 1, the control mice spent significantly more time with stranger 1 as compared to the empty cage (*t*-ratio = −2.43, *DF* = 9, *p* = 0.04; Figure 3B). However, the PB+PER exposed mice showed a lack of social preference spending an equal amount of time with either the empty cage or the stranger 1 (*t*-ratio = −0.09, *DF* = 11, *p* = 0.93; Figure 3B). When examining Social Novelty, the number of visits to either stranger 1 (familiar mouse) or stranger 2 (novel mouse), the control mice did not indicate a strong preference for one or the other (*t*-ratio = −1.05, *DF* = 9, *p* = 0.32; Figure 3C), similarly, no differences in social novelty were evident when examining PB+PER exposed (*t*-ratio = 0.61, *DF* = 9, *p* = 0.56). In addition, when examining social memory, as indicated by the time spent with either stranger 1 or stranger 2, the control mice appeared to spent slightly more time with the stranger 2, however, those differences did not reach statistical significance (*t*-ratio = 0.61, *DF* = 9, *p* = 0.56; Figure 3D), the PB+PER exposed mice did not show a strong preference for the novel mouse (stranger 2) over the familiar mouse (stranger 1) (t ratio = −0.06, *DF* = 11, *p* = 0.95). When examining Social Interaction—Proximity, by examining the time spent in the proximal zones, the control mice showed a statistically significant preference to spent more time with stranger 1 as compared to the empty cage (*t*-ratio = −1.85, *DF* = 9, *p* = 0.05; Figure 3E), demonstrating normal healthy social behaviors. However, the PB+PER mice exhibited a lack of social preference, demonstrated by a similar amount of time spent between stranger 1 and the empty cage (*t*-ratio = −0.19, *DF* = 11, *p* = 0.85; Figure 3E). When examining Social Memory—Proximity, the control mice showed a trend to spent more time with stranger 2 as compared to stranger 1, indicative of normal healthy social behaviors, although those differences did not reach statistical significance (*t*-ratio = 1.29, *DF* = 9, *p* = 0.23; Figure 3F). However, the PB+PER mice once again showed a lack of social preference, demonstrated by an similar amount of time spent between stranger 1 and stranger 2 (*t*-ratio = −0.36, *DF* = 11, *p* = 0.73; Figure 3F). Overall, the PB+PER exposed mice showed a lack of social interaction and social preference as demonstrated by increased disinterest in exploring and/or interacting with the novel mice at 13 months post exposure to GW agents (Figures 3A–F).

**Figure 4:**
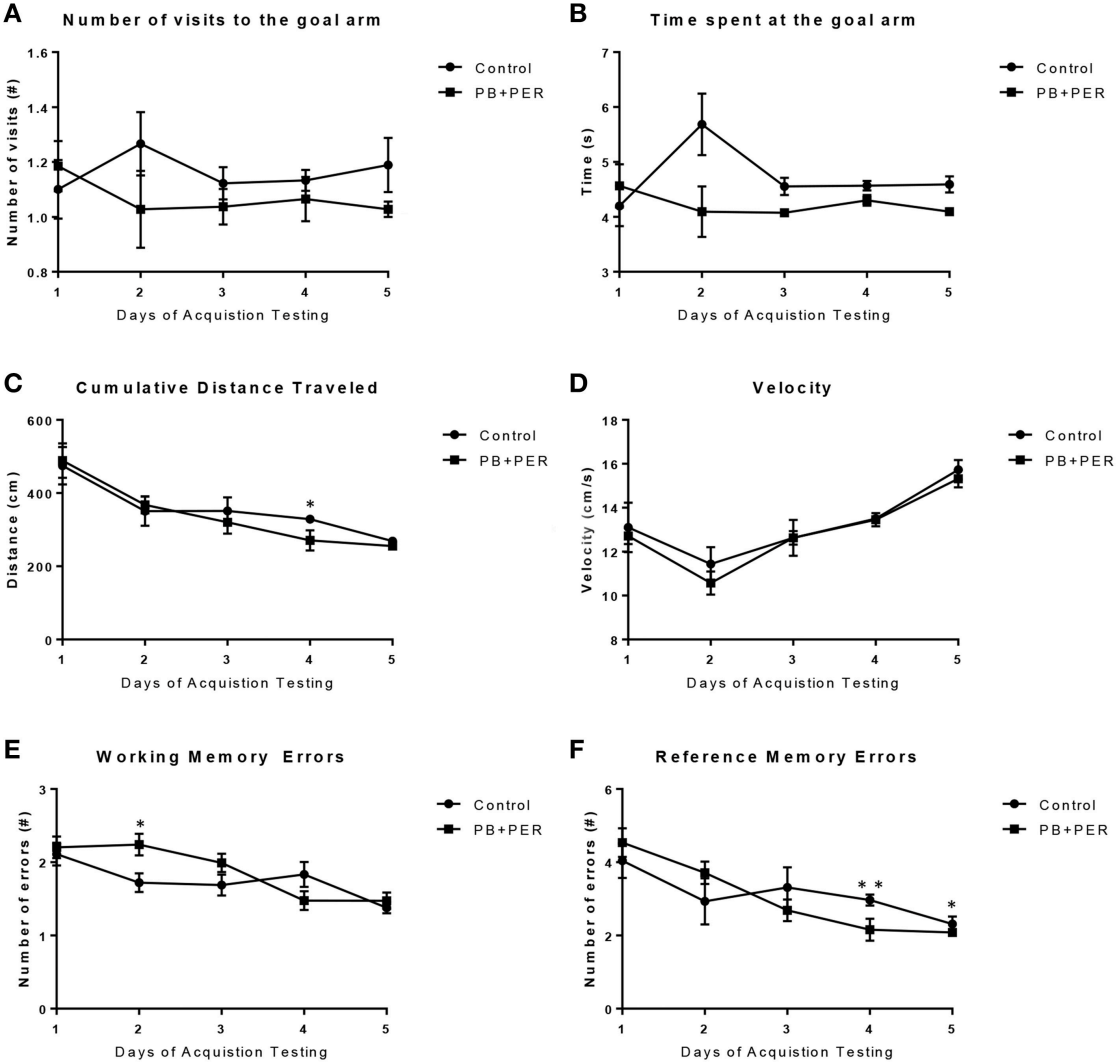
**Acute exercise may improve working and reference memory in exposed mice during RAWM acquisition testing, 13 months post exposure**. When examining **(A)** the Goal Arm Frequency (#), the number of times the mice entered the goal arm, and **(B)** the duration at the goal arm (s), no overall differences were observed between control and PB+PER mice. When examining **(C)** the cumulative distance traveled, a difference was noted on Day 4 between control and PB+PER mice, where PB+PER mice outperformed the control mice. No differences were noted when examining their **(D)** velocities by day. In addition, when examining **(E)** the number of working memory errors made by either the exposed mice or the control mice over the 5 day testing period at 13 months post exposure, differences were observed on day 2, where PB+PER mice made more errors as compared to controls. However, PB+PER exposed mice seemed to improve (make less errors) as they continued to advance through the acquisition days **(E,F)**. When examining **(F)** the number of reference memory errors, significant differences were observed between the two groups on days 4 and 5, where PB+PER mice performed better/on par with their control counterparts. Overall these data suggest that learning abilities are intact in PB+PER exposed mice, and that acute exercise may improve working and reference memory in PB+PER exposed mice. ^*^p < 0.05; ^**^p < 0.01.

The RAWM was used to investigate working and reference memory. When examining the Goal Arm Frequency (#), the number of times the mice visited the goal arm, a small main effect of exposure was observed (*F* = 4.39, *DF* = 1, *p* = 0.037; Figure 4A), where the exposed mice entered the goal arm less times as compared to controls, however, no statistically significant differences were observed when examining the interaction effect between exposure and days post-exposure (*F* = 0.84, *DF* = 14, *p* = 0.63). In addition, *post-hoc* testing revealed that there were no statistically significant differences between the control and PB+PER mice when they were examined by day (Supplementary Table 2). When examining the duration at the goal arm [time spent at the goal arms (s)], no main effect of exposure was observed when examining the differences between PB+PER mice as compared to controls (Wald X^2^ = 1.99, *DF* = 14, *p* = 0.16), however, an interaction effect between exposure and days post-exposure was noted (Wald X^2^ = 49.98, *DF* = 14, *p* < 0.001) where exposed mice spent less time at the goal arm(s) as compared to controls (Figure 4B). *Post-hoc* testing revealed that there were no statistically significant differences between the control and PB+PER mice when they were examined by day (Supplementary Table 2). Furthermore, when examining the cumulative distance traveled, no differences were detected between the two groups (*F* = 1.217, *DF* = 1, *p* = 0.271; Figure 4C). No differences were noted when examining their velocities (*F* = 0.616, *DF* = 1, *p* = 0.434; Figure 4D). *Post-hoc* testing revealed that there was a significant difference between the control and PB+PER mice on Day 4, where the PB+PER mice traveled a shorter distance as compared to the control mice (*p* = 0.04; Supplementary Table 2). No differences were noted when examining their velocities (*F* = 0.616, *DF* = 1, *p* = 0.434; Figure 4D), likewise, *post-hoc* testing revealed no differences between control and PB+PER mice when examining them by day (Supplementary Table 2). In addition, when examining the number of working memory errors made by either the exposed mice or the control mice over the 5 day testing period at 13 months post exposure (Figure 4E), no main effect of exposure was observed (Wald X^2^ = 0.51, *DF* = 1, *p* = 0.48), no statistically significant differences were observed when examining the interaction effect between exposure and days post-exposure (Wald X^2^ = 8.76, *DF* = 4, *p* = 0.06), however, a main effect of post-exposure days was observed (Wald X^2^ = 24.55, *DF* = 4, *p* < 0.001). *Post-hoc* testing revealed that there was a significant difference between the control and PB+PER mice on Day 2, where PB+PER mice made more working memory errors as compared to controls (*p* = 0.03; Supplementary Table 2). When examining the number of reference memory errors made by either the exposed or the controls over the 5 day testing period (Figure 4F), no main effect of exposure was observed (Wald X^2^ = 2.5, *DF* = 1, *p* = 0.12), a statistically significant difference was observed when examining the interaction between exposure and days post-exposure (Wald X^2^ = 16.45, *DF* = 4, *p* = 0.002), and a main effect of post-exposure days was observed (Wald X^2^ = 60.57, *DF* = 4, *p* < 0.001). *Post-hoc* testing revealed that there was a significant difference between the control and PB+PER mice on Day(s) 4 and 5, where the PB+PER mice outperform the control mice (Day 4, *p* = 0.01; Day 5, *p* = 0.03; Supplementary Table 2). Overall these data suggest that although learning abilities appear to be intact in PB+PER exposed mice, some discrete differences were observed in working and reference memory errors when examining the two groups by acquisition trial days, at 13 months post exposure to GW agents.

**Figure 5:**
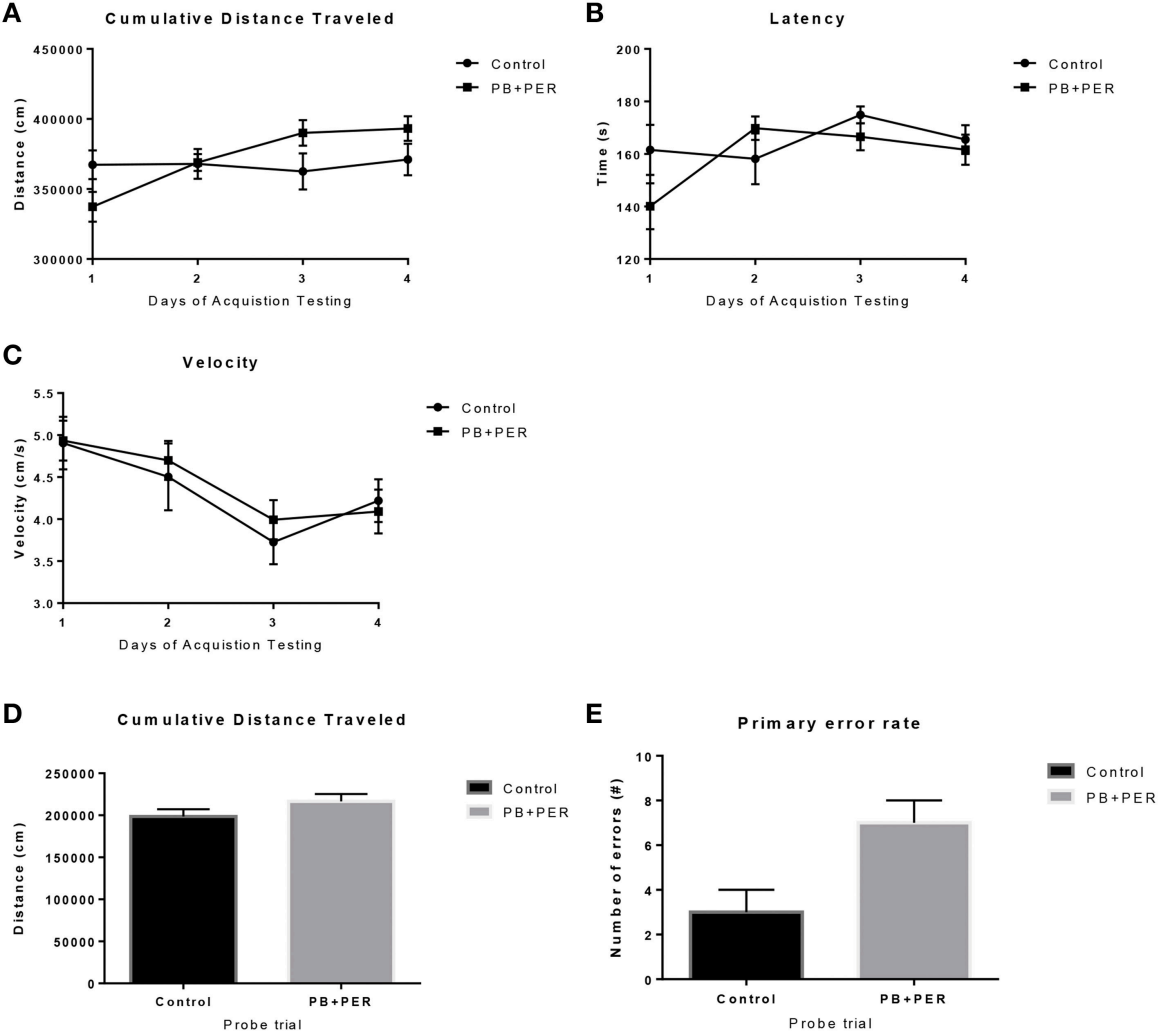
**No overall behavioral differences were observed in exposed mice during BM testing at 22.5 months post-exposure to PB+PER**. Control and exposed mice behaved similarly when we examined **(A)** cumulative distance traveled to the target hole, **(B)** escape latency, and **(C)** velocity, over a 4-day period. In addition, when the mice were examined 24-h after the last acquisition trial, by a single probe (day 5), the PB+PER exposed mice appeared to travel a longer **(D)** cumulative distance to reach the TH as compared to controls. When examining the **(E)** primary error rate (#), PB+PER exposed mice appeared to make more mistakes as compared to controls. Although there were apparent trends, these differences failed to reach statistical significance.

**Figure 6:**
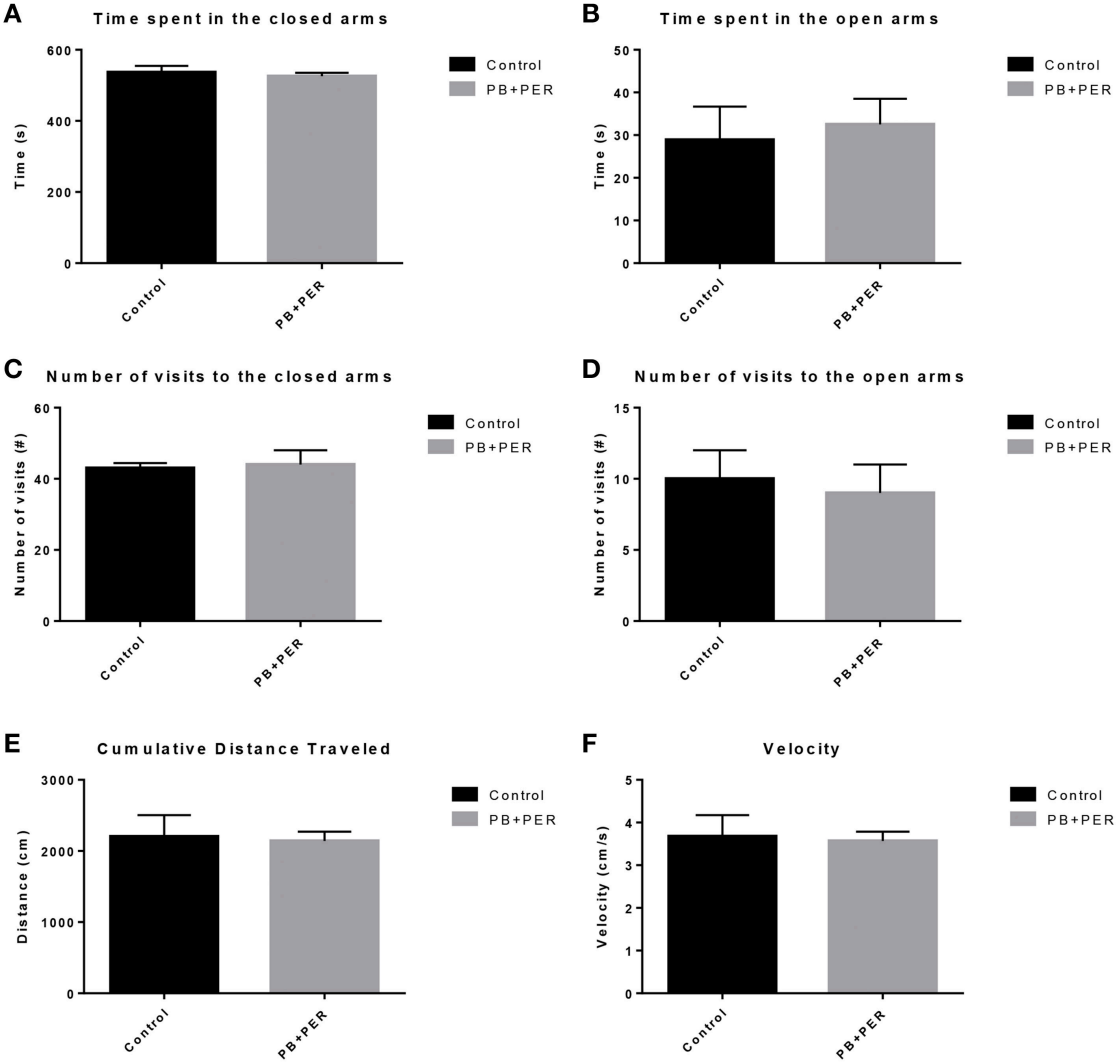
**No anxiety-like behaviors were observed in the PB+PER exposed mice, 22.5 months post exposure, as revealed by EPM testing**. EPM testing revealed that PB+PER exposed mice spent a similar amount of time in **(A)** the closed arms, and a similar amount of time in the open arms **(B)** as compared to their controls. No differences were noted between the two groups when examining **(C)** number of visits to the closed arms (#) and **(D)** the number of visits to the open arms. No differences were noted between the two groups when examining **(E)** cumulative distance traveled (cm) and **(F)** velocity (cm/s).

**Figure 7:**
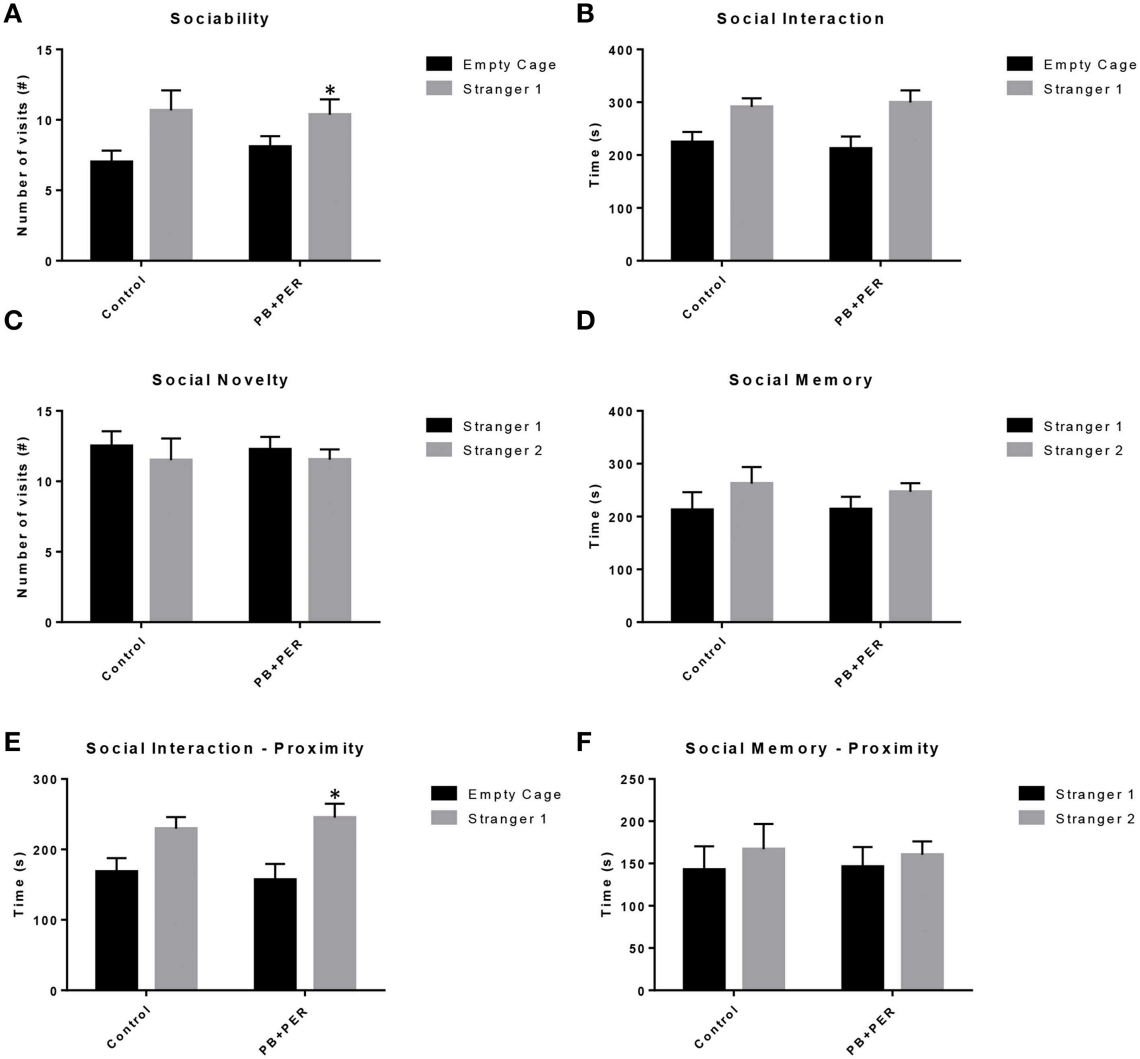
**Three Chamber testing revealed that PB+PER exposed mice exhibit normal sociability and social interaction behaviors, 22.5 months post exposure to GW agents**. When examining **(A)** Sociability, the number of visits to either the empty cage or stranger 1, the control mice exhibited a slight trend toward an increased number of visits to the stranger 1 mouse as compared to the empty cage. In addition, PB+PER exposed mice demonstrated preference for the novel mouse (stranger 1) over the empty cage. When examining **(B)** Social Interaction, the time (s) the animals spent with either the empty cage or stranger 1, the control mice appeared to spend more time with stranger 1 as compared to the empty cage, although those differences did not reach statistical significance. Similarly, the PB+PER exposed mice continued to demonstrate a clear preference for the novel mouse (stranger 1) as compared to the empty cage. When examining **(C)** Social Novelty, the number of visits to either stranger 1 (familiar mouse) or stranger 2 (novel mouse), the control mice did not indicate a strong preference for one or the other, likewise, no differences in social novelty were evident when examining PB+PER exposed mice. In addition, when examining **(D)** Social Memory, as indicated by the time spent with either stranger 1 or stranger 2, the control mice did not demonstrate a strong preference to spend more time stranger 2 over stranger 1. Likewise, the PB+PER exposed mice did not show a strong preference for the novel mouse (stranger 2) over the familiar mouse (stranger 1). When examining **(E)** Social Interaction—Proximity (s), the control mice showed a trend indicating increased preference to spent more time with stranger 1 as compared to the empty cage, demonstrating normal healthy social behaviors. Likewise, the PB+PER mice showed a statistically significant preference to spent more time with stranger 1 as compared to the empty cage. When examining **(F)** Social Memory—Proximity (s), both the control and the PB+PER exposed mice did not show a strong preference for the novel mouse (stranger 2) over the familiar mouse (stranger 1).

The same cohort of mice was assessed additionally by neurobehavioral testing at 22.5 months post exposure (~26 months of age). No overall behavioral differences were detected by BM testing between control and exposed mice, specifically when examining the cumulative distance traveled (cm) (*F* = 0.30, *DF* = 1, *p* = 0.59), escape latency (s) (Day 1: X^2^ = 3.47, *DF* = 1, *p* = 0.06; Day 2: X^2^ = 2.67, *DF* = 1, *p* = 0.10; Day 3: X^2^ = 0.03, *DF* = 1, *p* = 0.85, Day 4: X^2^ = 0.52, *DF* = 1, *p* = 0.47), and velocity (cm/s) (*F* = 0.07, *DF* = 1, *p* = 0.79) over 4 consecutive days of acquisition trials (Figures 5A–C). When examining short-term memory differences via probe trial 24 h after the last acquisition trial, there was a trend in the cumulative distance traveled, where exposed mice traveled a longer cumulative distance to reach the TH as compared to controls, although these differences failed to reach statistical significance (*F* = 2.20, *DF* = 1, *p* = 0.16; Figure 5D). Furthermore, when examining the number of primary error rate entries, exposed mice made more mistakes as compared to controls. Although there were apparent trends, these differences failed to reach statistical significance (*F* = 2.77, *DF* = 1, *p* = 0.13; Figure 5E). Likewise, no differences in velocity (cm/s) were observed during probe trial testing (*F* = 0.23, *DF* = 1, *p* = 0.64; data not shown). Additionally, anxiety-like behaviors were reassessed at this time point using the EPM. EPM testing revealed that PB+PER exposed mice spent a similar amount of time in the closed arms (*F* = 0.35, *DF* = 1, *p* = 0.56; Figure 6A), and a similar amount of time in the open arms (*F* = 0.12, *DF* = 1, *p* = 0.73; Figure 6B) as compared to their controls. No differences were noted between the two groups when examining the number of visits to the Closed Arms (#) (*F* = 0.06, *DF* = 1, *p* = 0.81; Figure 6C) and the number of visits to the Open Arms (*F* = 0.12, *DF* = 1, *p* = 0.73; Figure 6D). No differences were noted between the two groups when examining the cumulative distance traveled (cm) (*F* = 0.05, *DF* = 1, *p* = 0.82; Figure 6E) and velocity (cm/s) (*F* = 0.06, *DF* = 1, *p* = 0.82; Figure 6F). When examining Sociability, the number of visits to either the empty cage or stranger 1, the control mice exhibited a slight trend toward an increased number of visits to the stranger 1 mouse as compared to the empty cage (*t*-ratio = −2.23, *DF* = 5, *p* = 0.08; Figure 7A), although it did not reach statistical significance, it did demonstrate normal behaviors, demonstrating a preference for the novel mouse (stranger 1). In addition, PB+PER exposed mice demonstrating preference for the novel mouse (stranger 1) over the empty cage (*t*-ratio = −2.31, *DF* = 10, *p* = 0.04; Figure 7A). When examining Social Interaction, the time (s) the animals spent with either the empty cage or stranger 1, the control mice appeared to spend more time with stranger 1 as compared to the empty cage (*t*-ratio = −1.98, *DF* = 5, *p* = 0.10; Figure 7B), although those differences did not reach statistical significance. Likewise, PB+PER exposed mice continued to demonstrate a clear preference for the novel mouse (stranger 1) over the empty cage (*t*-ratio = −1.94, *DF* = 10, *p* = 0.08; Figure 7B). When examining Social Novelty, the number of visits to either stranger 1 (familiar mouse) or stranger 2 (novel mouse), the control mice did not indicate a strong preference for one or the other (*t*-ratio = −0.61, *DF* = 5, *p* = 0.57; Figure 7C), likewise, no differences in social novelty were evident when examining PB+PER exposed mice (*t*-ratio = −0.59, *DF* = 10, *p* = 0.57; Figure 7C). In addition, when examining Social Memory, as indicated by the time spent with either stranger 1 or stranger 2, the control mice did not demonstrate a strong preference to spend more time stranger 2 over stranger 1 (*t*-ratio = 0.78, *DF* = 5, *p* = 0.46; Figure 7D). Likewise, the PB+PER exposed mice did not show a strong preference for the novel mouse (stranger 2) over the familiar mouse (stranger 1) (*t*-ratio = 0.88, *DF* = 10, *p* = 0.40; Figure 7D). When examining Social Interaction—Proximity (s), the control mice showed a trend indicating increased preference to spent more time with stranger 1 as compared to the empty cage, demonstrating normal healthy social behaviors (*t*-ratio = −2.03, *DF* = 5, *p* = 0.10; Figure 7E). Likewise, the PB+PER mice showed a statistically significant preference to spent more time with stranger 1 as compared to the empty cage (*t*-ratio = −2.19, *DF* = 10, *p* = 0.05; Figure 7E). When examining Social Memory—Proximity (s), both the control (*t*-ratio = 0.48, *DF* = 5, *p* = 0.65; Figure 7F) and the PB+PER exposed mice (*t*-ratio = 0.42, *DF* = 10, *p* = 0.69; Figure 7F) did not show a strong preference for the novel mouse (stranger 2) over the familiar mouse (stranger 1).

### Neuropathological examination

**Figure 8:**
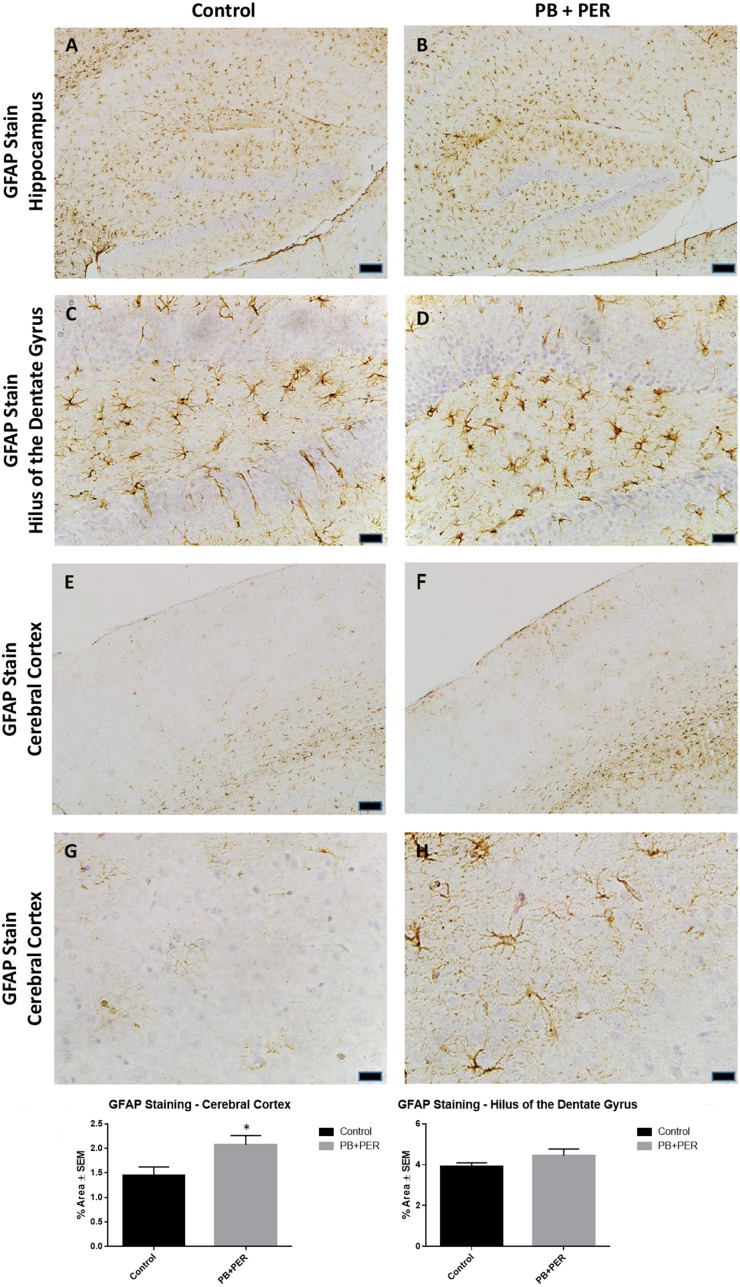
**PB+PER exposure altered astrocytic activation in the cerebral cortices of mice, 22.5 months post exposure**. PB+PER exposure did not significantly alter astrocytic activation in the hippocampi of exposed mice **(B,D)** compared to controls **(A,C)** at 22.5 months post exposure. PB+PER exposure significantly increased astrocytic activation in the cerebral cortices **(F,H)** of exposed mice, as compared to controls **(E,G)** at 22.5 months post exposure. Representative images used 10X **(A,B,E,F)**, and 40X **(C,D,G,H)** objectives (scale bars represent 100 and 20 μm, respectively). Histograms depict the quantification of the GFAP stain in the hippocampi and cerebral cortices from control and exposed mice, as % Area per microscopic field. ^*^p < 0.05.

**Figure 9:**
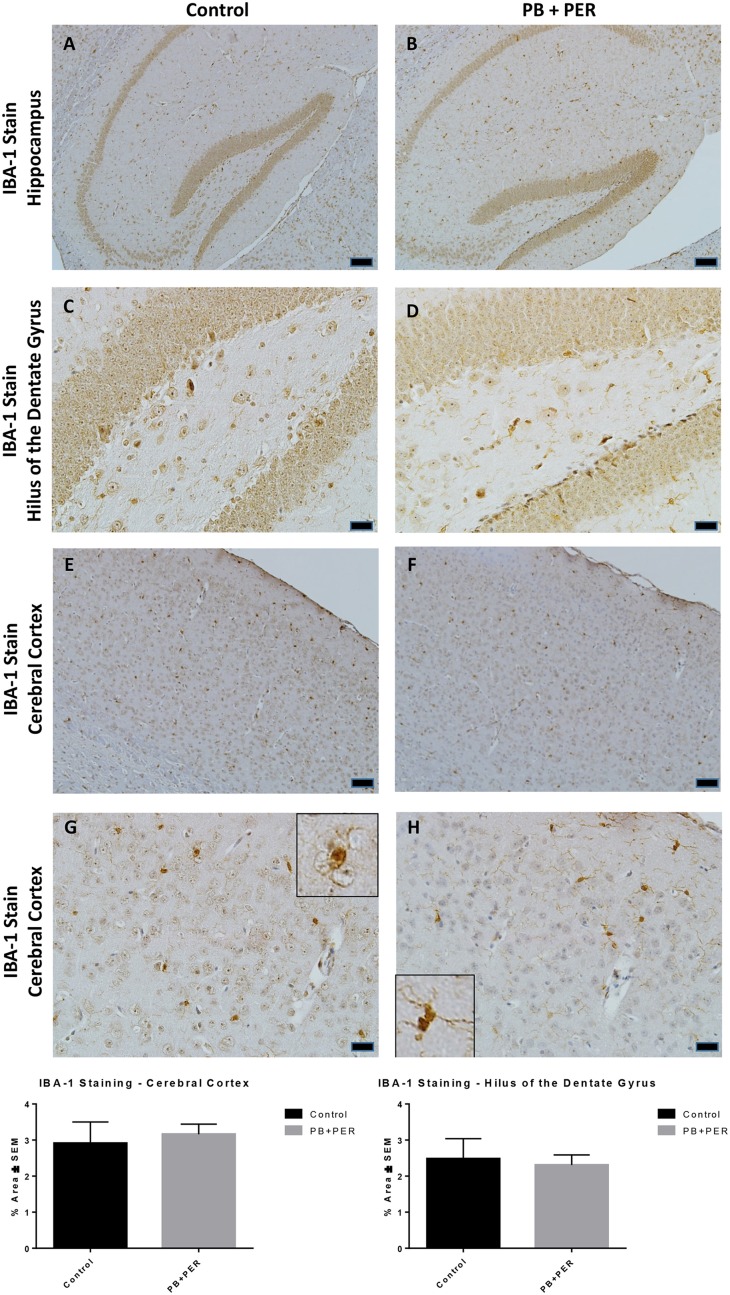
**PB+PER exposure did not alter microglial levels in hippocampi and cortices of mice, 22.5 months post-exposure**. The IBA-1 stain showed no differences between exposed **(B,D)** and control **(A,C)** mice in the hippocampi and the cerebral cortices of exposed **(F,H)** and control **(E,G)** animals. Insets in **(G)** and **(H)** represent magnified images of ramified and resting microglia in both control and PB+PER exposed mice, respectively, at 22.5 months post exposure to GW agents. Representative images used 10X **(A,B,E,F)**, and 40X **(C,D,G,H)** objectives, scale bars represent 100 and 20 μm, respectively. Histograms depict the quantification of the IBA-1 stain in the hippocampi and cerebral cortices from control and exposed mice as % Area per microscopic field.

**Figure 10:**
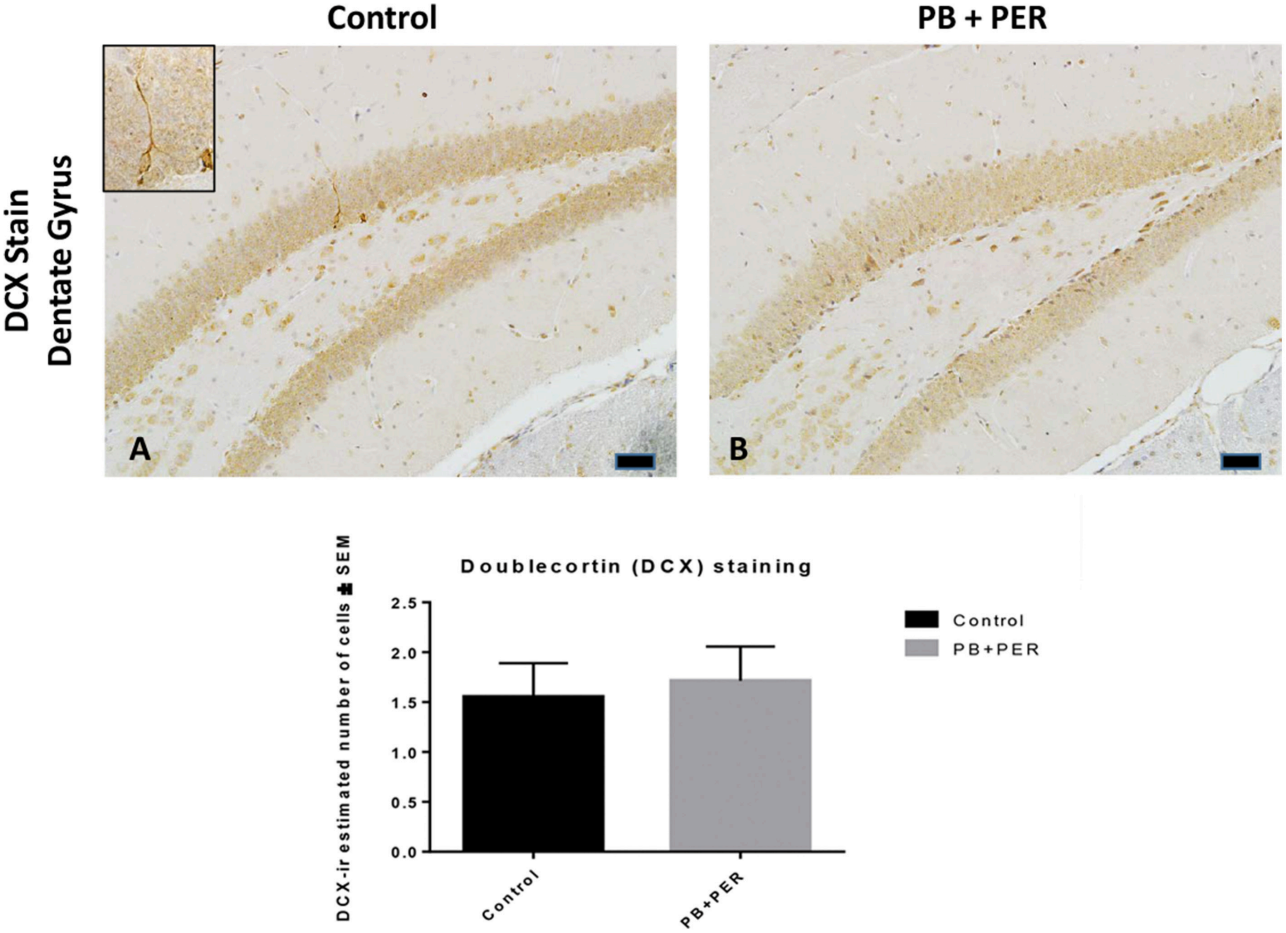
**No differences were observed in neurogenesis in mice, 22.5 months post exposure**. PB+PER exposure did not alter neurogenesis in the dentate gyrus (DG) region in exposed animals at 22.5 months post exposure **(B)** as compared to controls **(A)**. The depicted micrographs were taken using 20X **(A,B)**, scale bars represent 50 μm. Histograms depict the manual quantification of the doublecortin (DCX) stain in the dentate gyri, as total number of cells within the DG.

**Figure 11:**
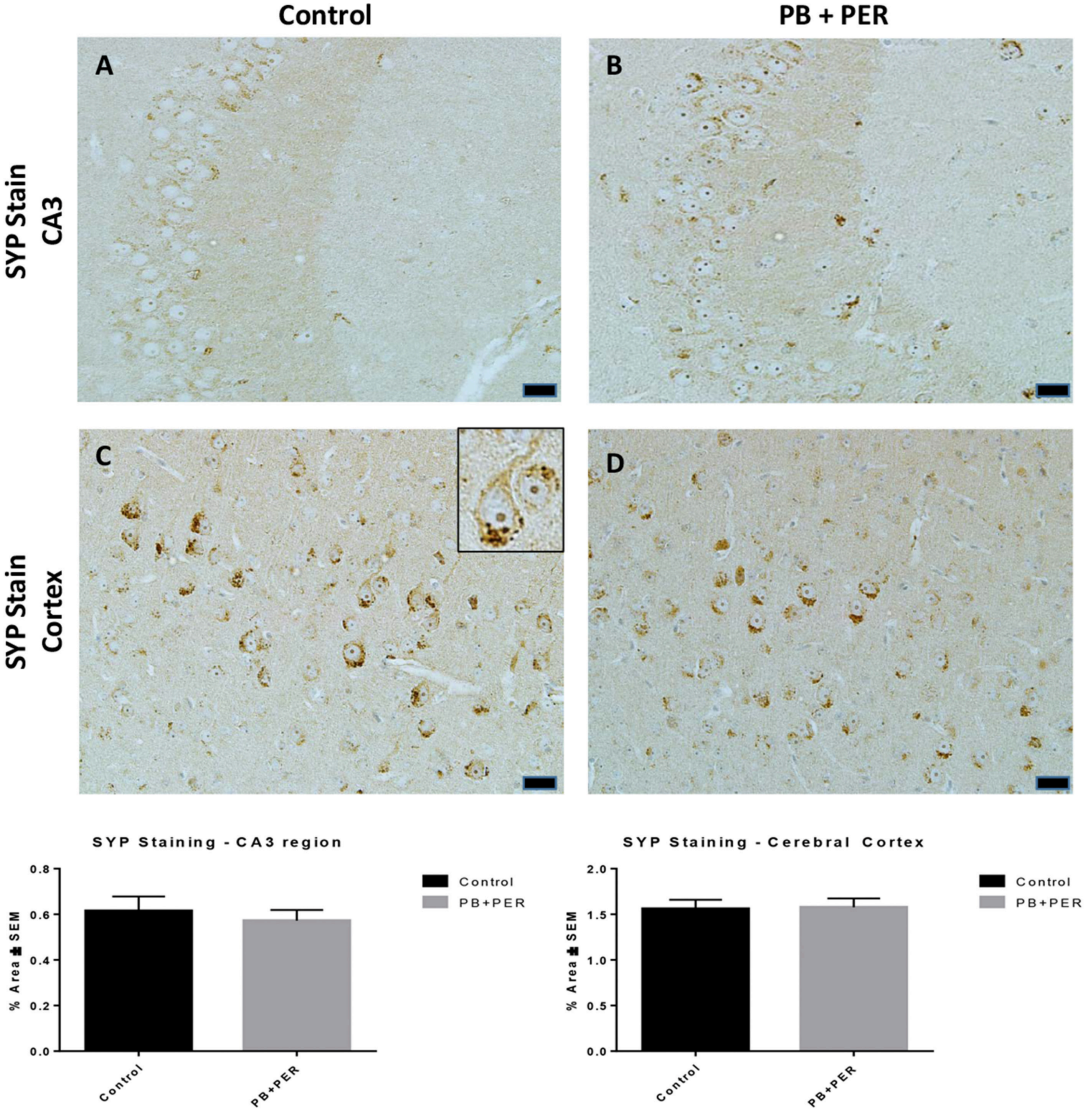
**No differences were observed in SYP staining in cerebral cortices and the CA3 regions of exposed mice, ~22.5 months post-exposure**. No differences were observed in SYP staining in the CA3 region of exposed mice **(B)** vs. controls **(A)**. No differences were observed in SYP staining in the cerebral cortices of exposed mice **(D)** vs. controls **(C)** (Student *t*-test, *p* = 0.84). Representative images were taken at 40X magnification (scale bars represent 20 μm). Inset depicts positive SYP staining showing dark brown pre-synaptic vesicles stained within the cell soma (see inset in **C**). Histograms depict the quantification of the SYP stain in the hippocampi and cerebral cortices, as % Area per microscopic field.

**Figure 12:**
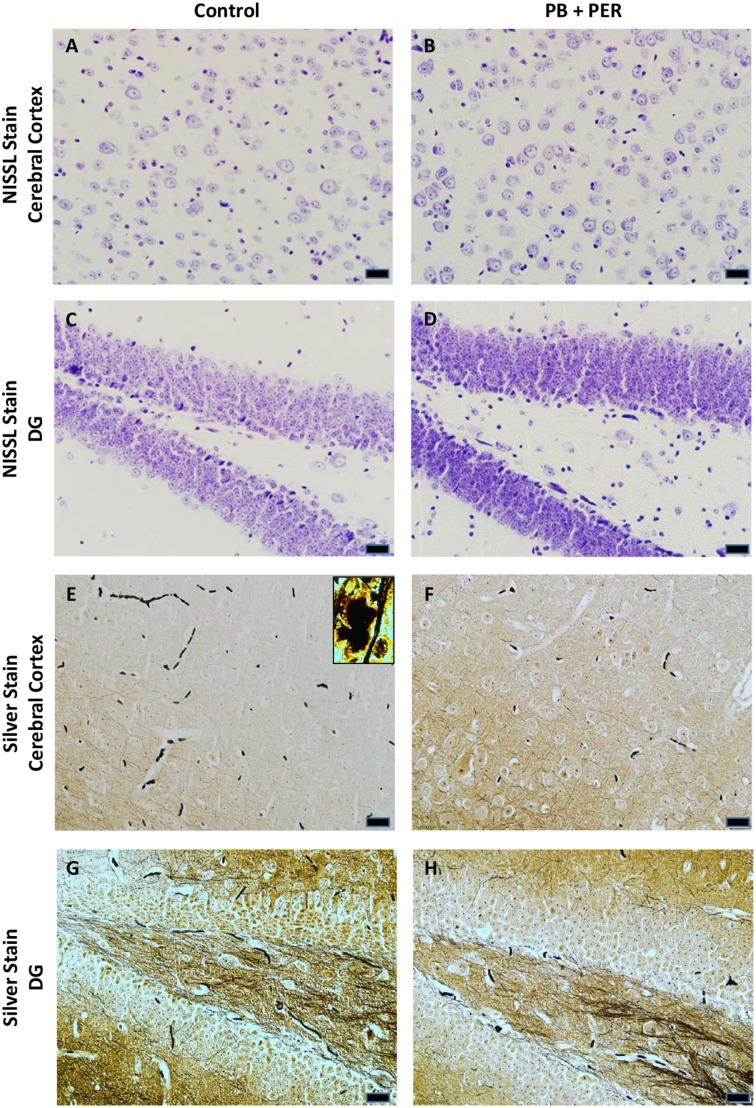
**No alterations in cell morphology detected 22.5 months post exposure to PB+PER**. Nissl staining revealed no gross morphological changes in nuclei/cell body of pyramidal neurons post exposure to PB+PER **(A–D)**. Similarly, the majority of cells in the hippocampi and cerebral cortices of PB+PER exposed mice **(F,H)** as compared to controls **(E,G)** were free from damaged and swollen axons and degenerated neurons when compared to a positive control (PSAPP mouse model of Alzheimer's Disease; see inset in **E**). Representative images were taken at 40X magnification (scale bar represents 20 μm).

At 22.5 months post exposure to PB+PER, swollen astrocytes were observed in the cerebral cortices of exposed animals as compared to controls as exemplified by increased GFAP staining (*F* = 5.79, *DF* = 1, *p* = 0.02; Figure 8). Although, a slight increase in GFAP staining in the hippocampi of PB+PER exposed mice as compared to controls was observed, those differences failed to reach statistical significance (*F* = 1.99, *DF* = 1, *p* = 0.17, Figure 8). No differences were observed in IBA-1 staining when examining the hippocampi (X^2^ = 0.69, *DF* = 1, *p* = 0.41) and cerebral cortices (X^2^ = 2.15, *DF* = 1, *p* = 0.14) of exposed mice vs. controls (Figure 9). No alterations in neurogenesis were observed in the dentate gyri of exposed mice as compared to controls (*F* = 0.15, *DF* = 1, *p* = 0.71; Figure 10). No noticeable changes in SYP staining were observed when examining the cerebral cortices (X^2^ = 0.02, *DF* = 1, *p* = 0.87), and the CA3 areas (X^2^ = 0.24, *DF* = 1, *p* = 0.62) of exposed mice compared to controls (Figure 11). Furthermore, no GW-agent induced alterations in neuronal cell morphology were observed in hippocampi or cerebral cortices (Figure 12). Similarly, the majority of cells in the hippocampi and cerebral cortices of PB+PER mice appeared to retain normal cell morphology and were free from damaged and swollen axons and degenerated neurons (Figure 12) when compared to a positive control (sagittal brain sections from the PSAPP mouse model of Alzheimer's disease).

## Discussion

GWI is a chronic multisymptom illness with a CNS component. The pathobiology of GWI remains to be fully elucidated, however, a growing body of evidence suggests that immune and inflammatory dysregulation may be a persistent feature of GWI (Rook and Zumla, 1997; Skowera et al., 2004; Peakman et al., 2006; Broderick et al., 2011, 2012, 2013; Smylie et al., 2013; Craddock et al., 2014; Johnson et al., 2014; O'Callaghan et al., 2015; O'Donovan et al., 2015). There is also a large body of evidence on the relation between exposure to pesticides and elevated rates of chronic diseases where inflammation is a major component. These include several types of cancers, diabetes, neurodegenerative disorders like Parkinson's disease, Alzheimer's disease, Amyotrophic Lateral Sclerosis (ALS), and recently, GWI (Bonetta, 2002; Sherer et al., 2002; Abdollahi et al., 2004; de Souza et al., 2011; Mostafalou and Abdollahi, 2013; Baltazar et al., 2014). The common feature of chronic disorders is a perturbation in cellular homeostasis, which can be induced via pesticides' primary mechanisms of action. These include disruption of ion channels, enzymes, and receptors (Mostafalou and Abdollahi, 2013). During the First Persian Gulf War, PB was used as a prophylactic agent against possible exposure to nerve gas agents such as sarin and soman (Sapolsky, 1998). The protective property of PB is due to its ability to shield the active site of the AChE from attack and subsequent irreversible inhibition by the nerve agents. PER is a broad-spectrum insecticide in the pyrethroid chemical family that works by quickly paralyzing the nervous systems of insects by interfering with the sodium channels. PER was employed as a pesticide by military personnel during the war, where uniforms and nets were pre-soaked with PER (Binns et al., 2008). Chronic neuroinflammation may be associated with chronic pain, fatigue, and cognitive impairment, and is recognized as one of the main symptom features of GWI in veterans of the Persian Gulf War (Fukuda et al., 1998; Steele, 2000; David et al., 2002; Vythilingam et al., 2005). GWI patients typically have altered pro- and anti-inflammatory cytokine expression in peripheral immune cells (Skowera et al., 2004; Broderick et al., 2011, 2013; Khaiboullina et al., 2014), and it has been suggested that exposure to PB and permethrin (PER) may have altered the balance of cytokine expression in veterans with GWI (Whistler et al., 2009; Broderick et al., 2011). PB and PER also modulate ACh-dependent immune mechanisms via inhibition of acetylcholinesterase (AChE) activity, through competitive and non-competitive mechanisms respectively, resulting in elevated peripheral ACh levels (Rao and Rao, 1995; Peden-Adams et al., 2004). GW agents have also been implicated in inducing immune responses similar to those seen with Th2 cell activation in the periphery via an ACh mediated mechanism (Punareewattana et al., 2001; Sullivan and Krieger, 2001; Nayak et al., 2004; Dantzer et al., 2008). Over-stimulation of the brain's immune response can shift it from homeostasis to a pro-inflammatory state which can result in deleterious effects on the central and peripheral nervous systems. These aberrant functions include nonspecific immune damage to neurons and impaired synaptic connections that accompany symptoms of cognitive impairment (Dantzer et al., 2008; Dilger and Johnson, 2008). To date, there are no effective treatments for GWI, and thus identification of biological pathways associated with long-term sequelae of exposure to GW agents is vital to understanding the pathogenic mechanisms of GWI and for developing novel therapies for treatment. Therefore, in this animal model of GW exposure, the consequences of combined PB+PER exposure in C57BL6/J mice were examined over a 2 year period, from 11 days to 22.5 months post exposure to GW agents. This work was conducted in order to set up a platform from which to identify biological mechanisms responsible for GW-agent induced pathobiology by spanning the life-time of the animal, so, we would be able to identify specific therapeutic targets in the future.

This study chronicles a longitudinal study in a mouse model of GWI by characterizing the chronic neurobehavioral and neuropathological outcomes following acute early life exposure to GW agents (PB+PER). A battery of neurobehavioral tests were conducted in a single cohort of mice from 11 days to 22.5 months post exposure to GW agents or vehicle control. At 11 days post exposure, no differences were observed in anxiety or any overt signs of locomotor impairment. However, at 13 months post exposure PB+PER exposed mice spent significantly larger proportion of time in the open arms(s) of the EPM, and had an increased number of visits to the open arms as compared to their controls. In addition, PB+PER exposed mice spent significantly larger proportion of time in the center (the junction between the closed and the open arms) of the EPM, as compared to their counterparts. These findings indicate disinhibited behavior in these mice, as a consequence of GW agent exposure, as mice normally preferentially spend more time in dark closed spaces. However, unlike our study, a recent study conducted by Parihar et al. (2013) using a rat model of GWI demonstrated increased anxiety related behavior after exposure to PB, PER DEET and stress at a less chronic time point post-exposure (3 months). In addition, anxiety-like features were observed in a different mouse model of GWI after 28 days of exposure to PB, PER, DEET, and stress, as indicated by an increase in time spent at the periphery of the OFT arena (Abdullah et al., 2012). Our studies are the first to demonstrate increased disinhibition in PB+PER exposed mice at 13 months after exposure. Disinhibition has been linked with dysfunction of the prefrontal cortex, an area which is crucial for decision making (Hains and Arnsten, 2008; Gruber et al., 2010). For example, Vasterling and colleagues have demonstrated a pattern of cognitive disinhibition and commission errors in a cohort of Persian Gulf War veterans in tasks which relate to attention and memory performance (Vasterling et al., 1998). However, with regards to GW agent exposure paradigms, further studies are warranted in order to definitively confirm the involvement of the prefrontal cortex in mediating anxiety-like behaviors seen in our model.

The Three Chamber Test and the RAWM were also performed at approximately 13 months post exposure. During the Three Chamber testing, PB+PER exposed mice showed a lack of social preference. Fiedler et al. reported that Gulf War Veterans (GWV) had a significantly higher prevalence of psychiatric diagnoses as compared to controls, with deployment as a powerful predictor of current depression and anxiety disorders decades after the end of the GW (Fiedler et al., 2006). Black et al. reported that GWV had a markedly higher prevalence of current anxiety disorders as compared to non-deployed military personnel (5.9 vs. 2.8%) (Black et al., 2004). Furthermore, anxiety disorders in GWV were associated with co-occurring psychiatric disorders such as panic disorder, generalized anxiety disorder and PTSD and in GWV as compared to controls were each present at rates nearly twice those expected (Black et al., 2004). Combat and extreme psychological stressors were less common and less sustained in the Gulf War as compared to other wars (including recent Middle East deployments), and PTSD rates are lower in GWV than in veterans of other wars (Binns et al., 2008). However, in a recent study of GWV, with and without PTSD, those with the co-morbid expression of PTSD showed increased brain activity in areas spanning the amygdala and the anterior cingulate cortex (Bierer et al., 2015). Since the anterior cingulate cortex has been associated with functions related to rational cognition, reward anticipation, decision making, impulse control and emotion (Bush et al., 2000, 2002; Williams et al., 2004), any change within this structure may lead to functional changes, such as behavioral disturbances.

Cognitive deficits using this PB+PER paradigm have been previously demonstrated in this model of GW agent at 5 months post exposure (~8.5 months of age) by Zakirova et al. (2015), therefore, as we wanted to expand the characterization of this mouse model, neurobehavioral testing in this cohort was conducted at much later time points. In addition, in this cohort we explored different mazes such as the RAWM and the BM, that are primarily designed to measure place learning and memory using environmental visuospatial cues. At 13 months post exposure, *post-hoc* testing revealed no significant differences between PB+PER exposed and control mice by day during RAWM testing when examining goal arm frequency [visits to the goal arm (#)] and goal arm duration [time spent at the goal arm (s)]. When examining the cumulative distance traveled, differences were detected on day 2 of acquisition trial by *post-hoc* testing, however, no overall differences were detected between the two groups; likewise, no differences were noted when examining their velocities. In addition, differences between exposed and control mice were detected using RAWM testing when examining working memory errors (#) and reference memory errors (#) made by either the exposed or control mice over the 5 day acquisition testing period. No main effect of exposure was observed when examining both parameters, although, significant differences were noted when examining the interaction between exposure and days post exposure for the number of reference memory errors (#). Additionally, apparent trends were noted in the exposed mice when examining both the number of errors made when examining both working and reference memory. Specifically, it appears as though during the first 3 days of RAWM acquisition testing the exposed mice behave poorly as compared to controls when examining working memory errors, however, by day 4 and 5 of acquisition testing the exposed mice perform better and/or on par with their controls littermates. Interestingly, similar trends are observed when examining the number of reference memory errors. Albeit, purely speculative, we suspect that this acute period of exercise may have improved and/or augmented the working and reference memory performance in PB+PER exposed mice. Exercise has been shown to improve memory acquisition and retrieval in mice (Van der Borght et al., 2007). In addition, exercise has been shown to significantly increase the number of maturing neurons, indicating that an increase in neurogenesis may be linked to the beneficial effects of exercise (Van der Borght et al., 2007). Furthermore, an acute period of exercise combined with working memory training has been shown to have synergistic and long-lasting effects on general cognitive performance in mice when voluntary running wheel access was combined with radial arm maze testing (Smith et al., 2013). Therefore, these data may indicate that acute exercise in the form of RAWM acquisition testing may have beneficial but transient effects on both working and reference memory in PB+PER exposed mice. Overall these data suggest that although learning abilities appear to be intact in PB+PER exposed mice, although some discrete differences in working and reference memory errors were observed when examining the two groups by acquisition trial days, at 13 months post exposure to GW agents.

It is also worth to consider that different maze tasks are able to measure different types of cognitive tasks; therefore, the fact that we used RAWM testing instead of BM testing, which in principal are both designed to measure place learning and memory using environmental visuospatial cues, harbor their own inherent differences. For instance, as Hodges (1996) highlighted, maze tasks differ from one another in many different ways: (a) the types of apparati, which may vary in environmental settings e.g., water vs. land) to multifaceted routes (such as the RAWM); (b) to different types of visuospatial, associative or sensory cues; (c) to various task requirements, which range from random search strategy/exploration to complex and structured sequences of choices; and (d) motivation, such as the opportunity for escape, or find shelter, or to explored novel objects in a new location. Given this multiplicity, it is likely that mazes showcase a variety of neuronal processes that may contribute to spatial learning and memory. Thus, the cognitive skill sets measured using one behavioral test may not be the same as those employed in another, which may create difficulties for the interpretation of exposure-related deficits. In addition, age plays an important role in mice in the context of age sensitivity as it relates to discerning exposure-dependent changes (Kennard and Woodruff-Pak, 2011). For example, Gower and Lamberty (Gower and Lamberty, 1993) reported that deficits in acquisition and retention of spatial memory are independent of spontaneous locomotor, sensorimotor, or emotional deficits from middle age (11–12 months) to old age mice (22 months). It has also been demonstrated that genotype has an effect on certain aspects of behavioral tests. For instance, Owen et al. (1997) conducted an extensive comparison of water maze testing performance in 12 mouse strains commonly used for genetic backgrounds and seven hybrid strains. Of those examined, only the C57BL6/J, C57BL10/J, and 129/SvevTac strains were capable of complex learning across multiple tasks, which included water maze testing. The C57BL6/J mice used in these studies were approximately 16.5 months of age and roughly 13 months post exposure to GW agents at the time of RAWM testing. Therefore, the behavioral differences observed in the PB+PER exposed mice during the RAWM acquisition testing, revealing alterations in working memory errors as well as reference memory errors exemplified by poor retention procedural aspects of the behavioral test, as well as poor retention of spatial memory, which are rely on both trial dependent and trial independent storage/processing of memories.

It is important to note that water maze based tests do not only highlight hippocampal-dependent tasks. Woodruff-Pak et al. demonstrated that the process of aging impacts brain structures and associated behaviors differentially, with the cerebellum showing earlier senescence than the hippocampus (Woodruff-Pak et al., 2010). Therefore, age may affect other brain regions, which may initiate age-related deficits in spatial memory performance. The BM is a hippocampal-dependent behavioral test (Yuede et al., 2007; Ziehn et al., 2010). In studies using unexposed, C57Bl6/J mice, older mice (12 and 18 months) made more errors during BM testing than younger mice (3 and 6 months) and relied more on a serial search strategy rather than a spatial strategy (Bach et al., 1999; Kennard and Woodruff-Pak, 2011). we have shown deficits in C57BL/6 mice as early as ~8.5 months of age and ~5 months post exposure to GW agents (Zakirova et al., 2015). In addition, when these GW agent exposed mice were tested at 26 months of age (22.5 months post exposure) using the BM, the exposed mice appeared to make more primary errors as compared to controls, although those differences failed to reach statistical significance. In addition, at this final time point of evaluation, no differences were observed in anxiety-like behaviors in PB+PER exposed mice as compared to controls when examined by EPM. Interestingly, Three Chamber testing at 22.5 months post exposure to GW agents revealed that PB+PER exposed mice exhibited normal sociability and social interaction behaviors, akin to the normal behaviors exhibited by their control counterparts. For instance when examining Sociability and Social interaction, the preference for visiting and spending time with either the empty cage or the novel mouse (stranger 1), PB+PER mice showed a strong preference for spending more time with the stranger 1 mouse over the empty cage, much like the control mice. However, when we examined Social Novelty and Social Memory, the preference for visiting and spending time with now familiar mouse (stranger 1) as compared to the novel mouse (stranger 2), both control mice and PB+PER mice did not exhibit a strong preference for spending time with one mouse over the other. Albeit purely speculative, these data may suggest that certain aspects of social memory/social novelty may be impaired in these mice at such an advanced age. Therefore, we hypothesize that an age effect masked the behavioral differences previously observed in these mice post exposure to GW agents.

The context of age sensitivity, as it relates to discerning exposure-dependent changes, is not only associated with neurobehavioral changes, but also correlates with neuropathological changes. Astrocytes undergo a complex age-dependent remodeling in a brain region-specific manner. The morphological aging of astrocytes was recently investigated in the cortices and the hippocampi of male SV129/C57BL6 mice of different age groups (3, 9, 18, and 24 months) (Rodríguez et al., 2014). This investigation revealed that GFAP-positive profiles in the hippocampus showed progressive age-dependent hypertrophy, as indicated by an increase in surface, volume, and somata volume at 24 months of age compared with 3-month-old mice (Rodríguez et al., 2014). On the other hand, aging induced a decrease in GFAP-positive astroglial profiles in the entorhinal cortex (EC) (Rodríguez et al., 2014). In contrast to these observations, an increase in GFAP immunostaining was observed in PB+PER exposed mice as compared to controls when examining their cerebral cortices 22.5 months post exposure. This increase in astrogliosis in the cerebral cortices of exposed mice is a constant feature of GW exposure in this animal model, which has been demonstrated at 5 months (Zakirova et al., 2015), 16 months (pers. comm., L. Abdullah), and 22.5 months post exposure (as mentioned here). In addition, there was a slight increase in GFAP staining in the hippocampi of PB+PER exposed mice as compared to controls at 22.5 months post exposure, however, those findings may have been confounded due to the age of the animals (26 months of age—close to the life-span for laboratory mice). Thus, these findings underscore astrogliosis as a persistent and pivotal feature of age-progressive cognitive impairments and neuropathological deficits as a result of GW agent exposure early on in life.

To the best of our knowledge, this work is the first to chronicle such an extensive chronic neurobehavioral characterization using an animal model of GW agent exposure. In addition, neuropathological studies were performed at 22.5 months post exposure to GW agents (well into the end of the life-span for laboratory mice). This lifespan analysis models, in mice, the time that has passed since the current GWI patient population received their pathogenic exposures (nearly two and half decades) as well as the expected progression of the illness, and thus is of considerable relevance for translational research.

In conclusion, the work detailed here describes the successful implementation of this model as a platform in which to identify biological mechanisms responsible for GW-agent induced pathobiology, and thereby to identify therapeutic targets. Validation of one of these targets will be done in the future studies. Specifically, given the persistent signature of chronic but mild neuroinflammation evident in this animal model, future studies will focus on implementing anti-inflammatory agents in order to investigate whether therapeutic intervention earlier in life (middle age) will be beneficial in mediating the effects of inflammation and thereby therapeutically modulating cognitive impairment in this mouse model of GW agent exposure.

## Author contributions

Conceived and designed the experiments: GA, FC, and VM. Performed the experiments: ZZ, SH, and LH. Analyzed the data: GC, LA, and ZZ. Contributed reagents/materials/analysis tools: FC and GA. Wrote the paper: ZZ, FC, and GA.

## Funding

This research was funded by a Congressionally Directed Medical Research Program award to GA (GW100076), VA merit award to FC and by the Roskamp Foundation. The funders had no role in study design, data collection and analysis, decision to publish, or preparation of the manuscript.

### Conflict of interest statement

The authors declare that the research was conducted in the absence of any commercial or financial relationships that could be construed as a potential conflict of interest.

## Supplementary material

Supplementary Figure 1 **Schematic illustrating the battery of neurobehavioral testing undertaken from 11 days to 22.5 months (684 days) post exposure to GW agents, PB+PER**.

Supplementary Table 1 **Statistical analysis of dependent variables examined during Open Field Testing, 11 days post exposure to GW agents, PB+PER**. Data was normally distributed, and therefore, One-way ANOVA analysis was used.

Supplementary Table 2 **RAWM *Post-hoc* analyses (multiple comparisons testing using Least Significant Difference)**.
