# Supplementary figures and images for: A Chronic Longitudinal Characterization of Neurobehavioral and Neuropathological Cognitive Impairment in a Mouse Model of Gulf War Agent Exposure
Source: Front Integr Neurosci. 2016 Jan 12;9:71. doi: 10.3389/fnint.2015.00071 (PMC4709860; doi:10.3389/fnint.2015.00071)

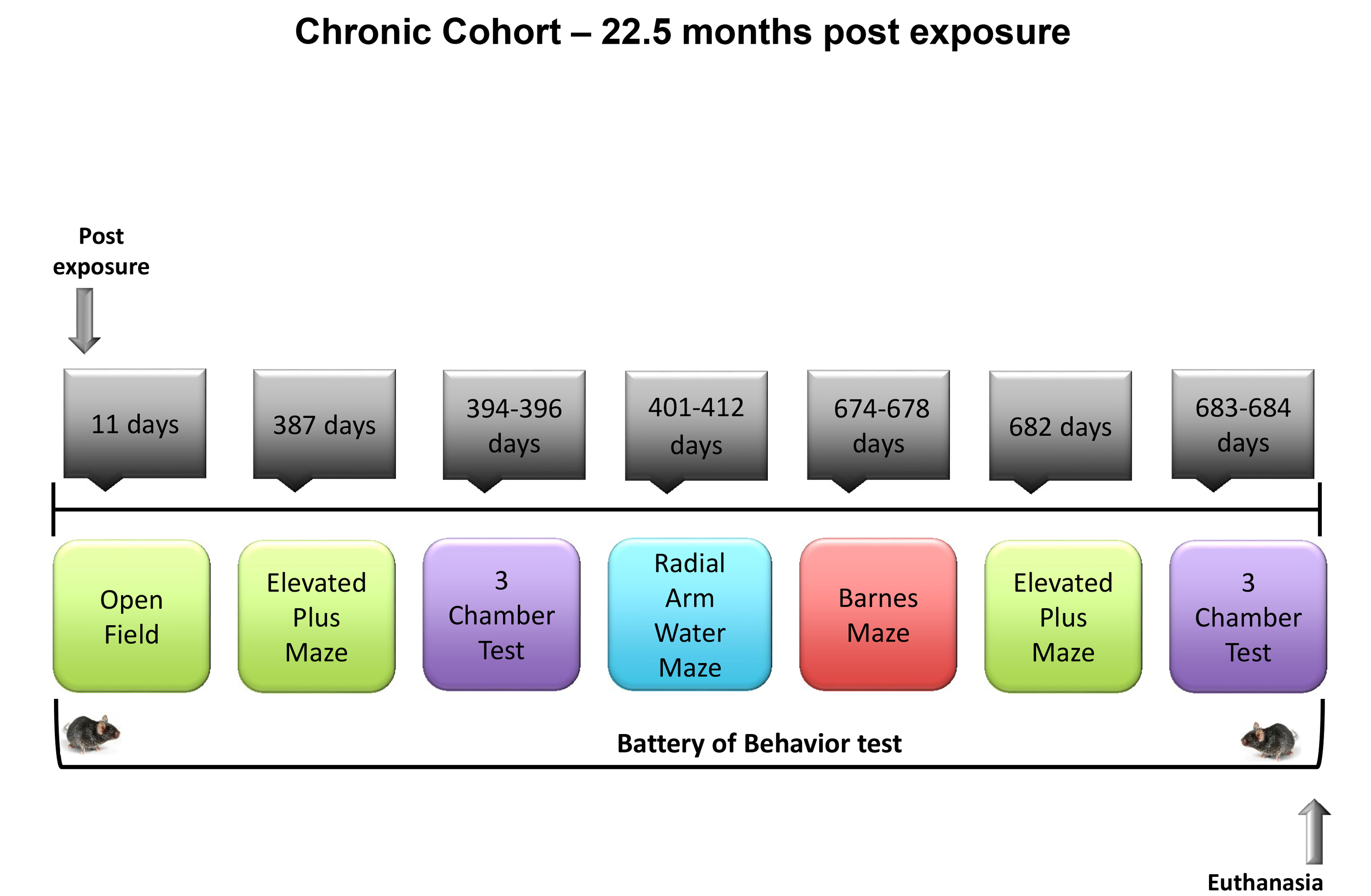

Supplement: Supplementary Figure 1 — Schematic illustrating the battery of neurobehavioral testing undertaken from 11 days to 22.5 months (684 days) post exposure to GW agents, PB+PER. [file Image1.TIF]
