# Supplementary material for: A Chronic Longitudinal Characterization of Neurobehavioral and Neuropathological Cognitive Impairment in a Mouse Model of Gulf War Agent Exposure
Source: Front Integr Neurosci. 2016 Jan 12;9:71. doi: 10.3389/fnint.2015.00071 (PMC4709860; doi:10.3389/fnint.2015.00071)
Supplement: Supplementary Table 1 — Statistical analysis of dependent variables examined during Open Field Testing, 11 days post exposure to GW agents, PB+PER. Data was normally distributed, and therefore, One-way ANOVA analysis was used. [file Table1.PDF]

Time (min)	Dependent Variable(s)	DF	Sum of Squares	F Ratio	Prob > F
Start-0:05:00	Cumulative Distance Moved (cm)	1	10966.3	0.0172	0.8974
0:05:00-0:10:00	Cumulative Distance Moved (cm)	1	200063.5	0.8447	0.3717
0:10:00-0:15:00	Cumulative Distance Moved (cm)	1	101592.1	0.321	0.5789
Start-0:05:00	Perimeter Duration (s)	1	828.1	1.4928	0.2395
0:05:00-0:10:00	Perimeter Duration (s)	1	576.4	0.8688	0.3651
0:10:00-0:15:00	Perimeter Duration (s)	1	117	0.1462	0.7072
Start-0:05:00	Mobile Duration (s)	1	494.8	0.4269	0.5228
0:05:00-0:10:00	Mobile Duration (s)	1	122.3	0.2347	0.6346
0:10:00-0:15:00	Mobile Duration (s)	1	66.9	0.1431	0.7102
Start-0:05:00	Immobile Duration (s)	1	490.6	0.4224	0.525
0:05:00-0:10:00	Immobile Duration (s)	1	122.3	0.2347	0.6346
0:10:00-0:15:00	Immobile Duration (s)	1	66.9	0.1431	0.7102
Start-0:05:00	Inner Circle Duration (s)	1	67.02305	0.4139	0.5291
0:05:00-0:10:00	Inner Circle Duration (s)	1	191.54134	0.502	0.4888
0:10:00-0:15:00	Inner Circle Duration (s)	1	8.69743	0.0228	0.8817
Start-0:05:00	Inner Circle Frequency (#)	1	5.04167	0.3048	0.5885
0:05:00-0:10:00	Inner Circle Frequency (#)	1	45.375	2.0242	0.174
0:10:00-0:15:00	Inner Circle Frequency (#)	1	22.04167	0.673	0.4241
